# Constructing eRNA-mediated gene regulatory networks to explore the genetic basis of muscle and fat-relevant traits in pigs

**DOI:** 10.1186/s12711-024-00897-4

**Published:** 2024-04-09

**Authors:** Chao Wang, Choulin Chen, Bowen Lei, Shenghua Qin, Yuanyuan Zhang, Kui Li, Song Zhang, Yuwen Liu

**Affiliations:** 1 Shenzhen Branch, Guangdong Laboratory for Lingnan Modern Agriculture, Key Laboratory of Livestock and Poultry Multi-Omics of MARA, Agricultural Genomics Institute at Shenzhen, Chinese Academy of Agricultural Sciences, Shenzhen, 518124 People’s Republic of China; 2 Innovation Group of Pig Genome Design and Breeding, Research Centre for Animal Genome, Agricultural Genomics Institute at Shenzhen, Chinese Academy of Agricultural Sciences, Shenzhen, 518124 People’s Republic of China; 3 Key Laboratory of Agricultural Animal Genetics, Breeding and Reproduction of Ministry of Education & Key Lab of Swine Genetics and Breeding of Ministry of Agriculture and Rural Affairs, Huazhong Agricultural University, Wuhan, 430070 People’s Republic of China; 4 School of Life Sciences, Henan University, Kaifeng, 475004 People’s Republic of China; 5 Shenzhen Research Institute of Henan University, Shenzhen, 518000 People’s Republic of China; 6 Kunpeng Institute of Modern Agriculture at Foshan, Chinese Academy of Agricultural Sciences, Foshan, 528226 People’s Republic of China

## Abstract

**Background:**

Enhancer RNAs (eRNAs) play a crucial role in transcriptional regulation. While significant progress has been made in understanding epigenetic regulation mediated by eRNAs, research on the construction of eRNA-mediated gene regulatory networks (eGRN) and the identification of critical network components that influence complex traits is lacking.

**Results:**

Here, employing the pig as a model, we conducted a comprehensive study using H3K27ac histone ChIP-seq and RNA-seq data to construct eRNA expression profiles from multiple tissues of two distinct pig breeds, namely Enshi Black (ES) and Duroc. In addition to revealing the regulatory landscape of eRNAs at the tissue level, we developed an innovative network construction and refinement method by integrating RNA-seq, ChIP-seq, genome-wide association study (GWAS) signals and enhancer-modulating effects of single nucleotide polymorphisms (SNPs) measured by self-transcribing active regulatory region sequencing (STARR-seq) experiments. Using this approach, we unraveled eGRN that significantly influence the growth and development of muscle and fat tissues, and identified several novel genes that affect adipocyte differentiation in a cell line model.

**Conclusions:**

Our work not only provides novel insights into the genetic basis of economic pig traits, but also offers a generalizable approach to elucidate the eRNA-mediated transcriptional regulation underlying a wide spectrum of complex traits for diverse organisms.

**Supplementary Information:**

The online version contains supplementary material available at 10.1186/s12711-024-00897-4.

## Background

Significant genetic and phenotypic diversity exists among breeds of domestic animals, which provide ideal models to understand the genetic basis of complex traits. Linking genetic variants to phenotypes using such models is critical in improving livestock production capacity and in elucidating the genetic basis of traits shared by humans and animals. In humans and animals, extensive efforts in mapping causal genetic variants that underlie traits suggest that more than 90% of the single nucleotide polymorphisms (SNPs) that are associated with various complex traits or diseases are located in the non-coding part of the genome [1–3]. Compared to the biological interpretation of coding variants, our lack of understanding of the genetic code of non-coding regulatory variants has motivated us to initiate several ambitious projects with the aim of systematically characterizing how the non-coding genome functions in various organisms. These projects include ModENCODE for model organisms [4], ENCODE for humans [5], and FAANG for farm animals [6]. These collaborative projects have not only generated multidimensional and temporo-spatial regulatory landscapes in different species, but have also played an important role in identifying the causal non-coding DNA variants and their target genes that would otherwise have been overlooked [7–11].

In spite of the extensive body of research in functional genomics conducted in humans and animals, there remains untapped potential in the comprehensive exploration of the eRNA landscape and the identification of crucial transcriptional regulatory networks mediated by eRNAs. These untapped resources hold great promise for advancing our understanding of the genetic basis of complex traits. Discovered in the 1980s, eRNAs are transcribed from enhancer regions [12], and their expression levels have been found to be strongly correlated with enhancer activity [13, 14]. In vivo, eRNA has multiple regulatory functions, such as mediating the formation of enhancer-promoter loops, maintaining an open chromatin state, changing the chromatin spatial conformation, and recruiting RNAP2 and other transcription initiation complex molecules [13, 15–17]. In recent years, the important biological functions of eRNAs have just began to be unraveled in the regulation of diseases, such as cancer, neurodegenerative diseases, cardiovascular diseases, and metabolic diseases [18]. Therefore, comprehensively analyzing the role of eRNAs, as a hub in integrating signaling from upstream transcription factors (TF) to regulate downstream target genes, would significantly contribute to understanding the genetic basis of complex traits.

The identification of eRNAs presents a significant challenge due to their inherently short length, infrequent splicing, and instability. In spite of these difficulties, the progress of sequencing technologies has provided several methods, such as global run-on sequencing (GRO-seq) [19], precision run-on sequencing (PRO-seq) [20], native elongating transcript sequencing (NET-Seq) [21], cap analysis gene expression and deep sequencing (CAGE-seq) [22], to identify eRNAs. Although efficient, the complex and costly nature of these methods remains a hindrance to their widespread use. An alternative approach is to use RNA-seq data to identify eRNAs [23–25]. There has been a growing trend of using this approach to uncover eRNAs in various biological contexts [26–30], including the first animal eRNA database with eRNA annotation in 10 species [31]. However, the eRNA annotation in pigs has yet to be established.

Among domesticated animals, the pig (*Sus scrofa*) holds a pivotal role, serving not only as a crucial source of protein and fat but also as an exceptional model for biomedical research. More importantly, geographical divergence, local adaptation, and artificial selection have resulted in significant phenotypic differences between eastern (Asia) and western (Europe and America) pigs, including lean meat mass and fat deposition [11, 32, 33]. In the pig husbandry industry, the pursuit of lean meat mass and controlled fat deposition stands as crucial breeding objectives, given their direct impact on meat quality and production efficiency. The Enshi Black (ES) pig, a representative of eastern pig breeds has a stronger fat deposition ability than the western breed Duroc pig. By comparison, the later has a higher lean meat ratio than the former. These contrasting phenotypes offer a unique perspective to study the genetic mechanisms that underlie the development and homeostasis of muscle and fat tissues.

In this study, our aim was to profile the landscape of eRNAs in the muscle and fat tissues of Duroc and ES pigs, respectively. We present potential hub eRNAs and their target genes by an integrative approach combining eRNA-mediated transcriptional regulatory networks, genome-wide association study (GWAS) signal and STARR-seq experiments. STARR-seq is a sequencing-based high-throughput method that allows a direct measure of the allelic regulatory activity of SNPs [34, 35]. Our study not only sheds light on the genetic regulatory mechanisms underpinning lean meat mass and fat deposition in pigs, but also proposes an integrative framework to pinpoint eRNA-mediated genetic regulatory networks that might substantially influence complex traits in various species.

## Methods

### Data source

In this study, we used H3K27ac ChIP-seq data obtained from the *longissimus dorsi* skeletal muscle and subcutaneous fat tissues of 2-week-old Duroc and ES pigs to identify enhancers, with two biological replicates for each breed. In addition, we integrated RNA-seq data from these pigs, covering various tissues such as skeletal muscle (*longissimus dorsi*), subcutaneous fat, heart, liver, and spleen, for a comprehensive quantitative analysis of gene and RNA expression. In addition, we included topologically associated domain (TAD) information for the *longissimus dorsi* skeletal muscle of two-week-old Large White pigs. All datasets were sourced from the research conducted in Professor Shuhong Zhao’s laboratory under the accession number PRJNA597497 [36]. To complement our investigation, we accessed GWAS hits and quantitative trait loci (QTL) regions associated with different traits in pigs from pigQTLdb, available at https://www.animalgenome.org/cgi-bin/QTLdb/SS/index. Furthermore, we collected GWAS summary statistics for 64 human complex traits from Hook’s study [37], accessible at https://doi.org/10.5281/zenodo.3253180. These datasets were selected based on their relevance to our research questions and their availability.

### Identification of enhancers and super enhancers

To obtain annotation information on genomic enhancers in the muscle and adipose tissue of Duroc and ES pigs, we acquired H3K27ac signal peaks for identifying the location of enhancers. Each tissue consisted of two biological replicates. In order to ensure consistency in the H3K27ac signal peaks within each tissue, we merged the peaks from the biological replicates using the ‘intersect’ and ‘merge’ commands in BEDTools (version 2.31.0) with the default parameters [38]. Subsequently, we excluded H3K27ac signal peaks that overlapped with the transcription start site (TSS) of known genes within ± 1 kb and considered the remaining H3K27ac peaks as enhancers. To measure the activity of enhancers in tissues, we downloaded the raw ChIP-seq data of the H3K27ac histone modification for evaluating enhancer activity. First, we processed the ChIP-seq data using the TrimGalore software (version 0.6.7, Babraham Institute, Cambridge, UK) to eliminate sequencing adapters, low-quality bases (Phred < 20), and short sequences (-q 20 --phred33 --stringency 4 --length 25 -e 0.1). After quality control, we aligned the clean data to the susScr11 genome using the Bowtie2 (version 2.4.4) software with the following parameters: --very-sensitive -X 1500 -x genome index -1 fq1 -2 fq2 [39]. PCR duplicates were removed using the Sambamba (version 1.0.0) [40] software. From each sample, we extracted pairs of aligned concordant reads, resulting in bam files for quantifying enhancer activity. Referring to Zhao's approach [36], we used the multiBamSummary BED-file function in deepTools [41] (v2.088) to count the number of reads within the ± 1 kb region around the center of different tissue enhancers. We then normalized the enhancer read counts by dividing them by the total number of reads in the library. Finally, we assessed the intensity of enhancer activity by calculating the fold-change value (IPRPM /INPUTRPM, where IPRPM represents the normalized signal strength of enhancers in the IP library, and INPUTRPM corresponds to the normalized signal strength of enhancer regions in the INPUT library). The dynamic activity heatmap depicting enhancer activity in fat and muscle tissue was created using the ‘Heatmap’ function from the ‘ComplexHeatmap’ R package (version 2.6.2) within the R software (version 4.0.5).

To identify super-enhancers (SE) in Duroc muscle, Duroc fat, ES muscle, and ES fat tissues, the bam files from biological replicates of each tissue were combined. Next, we used the MACS2 (version 2.2.8) [42] software to identify H3K27ac peaks specific to each tissue. Subsequently, with the resulting files and the genomic annotation file for the pig, we employed the ROSE (version 0.1) [43] software using default parameters to pinpoint the SE for each tissue.

### Identification of eRNAs

As the transcriptional range of eRNAs can be wider than the enhancer region [44], we expanded our enhancer set by ± 3 kb around the central point of each enhancer, delineating it as the potential transcriptional activity region of enhancers, in alignment with established methodologies from previous research [27, 29]. In order to mitigate interference from known coding genes or non-coding RNAs (such as miRNA, misc_RNA, rRNA, snoRNA, snRNA, tRNA, and lncRNA) during eRNA quantification, we followed the eRNA identification method outlined in Carullo et al. [30]. In the subsequent transcription signal quantification analysis, our exclusive focus was on the potential transcription regions of enhancers falling more than 1 kb outside of the genes and non-coding RNAs curated by RefSeq, UCSC, and Ensembl databases. Subsequently, we used strand-specific RNA-seq data from two biological replicates of muscle and adipose tissue in ES and Duroc pigs for eRNA analysis. The individuals contributing to the RNA-seq data were the same as those from which the H3K27ac ChIP-seq data were derived. Initially, quality control for the RNA-seq data was conducted using the TrimGalore software (version 0.6.7). The clean data were then mapped to the reference genome (susScr11) using the Hisat2 (version 2.2.1) software [45], using the (--rna-strandness RF) option for strand-specific mapping. Following the mapping step, we employed the Seqmonk software (Babraham Institute) in accordance with a previously described methodology [30] to assess the expression levels of eRNAs. In our study, we defined a mean RPM value of ≥ 1 in biological replicates specific to each tissue as the threshold for detectable eRNA expression in that tissue. In addition, we considered the directionality of eRNA transcription. If the proportion of reads mapped to the positive strand of the genome fell between 5 and 95% of the total reads mapped to the enhancer region, the eRNA was classified as bidirectionally transcribed and otherwise, as unidirectional transcribed. To visualize the distribution of detectable eRNAs across various tissues, we used the UpSetR (version 1.4.0) [46] package in R. In addition, we generated a heatmap of sample-to-sample distances using the expression values of detectable eRNAs, and the pheatmap() function from the pheatmap package (version 1.0.10).

### Analysis of eRNA characteristics

To compare the GC content of eRNAs with different transcriptional directions, we used the “nuc” command from the BEDTools software (v2.31.0) to calculate the GC content ratio within each eRNA sequence. Subsequently, we performed a Student's t-test to assess the differences in GC content between the groups of bidirectional and unidirectional eRNAs. The differences in expression level between unidirectional and bidirectional eRNAs, as well as between eRNAs within and outside SE, were evaluated using a two-sided unpaired Wilcoxon test. To explore the relationship between eRNA expression level and enhancer activity in each tissue, the detectable eRNAs were sorted from highest to lowest expression level and divided into eight bins. Then, we used the cor.test function in the R program (version 4.0.5) to assess the correlation between the mean eRNA expression levels and the mean enhancer activity across these bins (method = “pearson”). All the statistical analyses mentioned above were conducted using the R programming language. In our study, we conducted all gene ontology (GO) enrichment analyses using the enrichGO function from the R package clusterProfiler (version 3.14.3) [47]. Functional enrichment analysis of tissue-specific expression genes and eRNA target genes was directly performed using enrichGO. For eRNA-related functional annotation analysis, we extracted neighboring genes and performed the analysis.

### Deep learning model for prediction of enhancers with different transcription patterns

To analyze the sequence features of enhancers with different transcription directions, we used two prominent deep learning classification models: DeepSEA [48] and a convolutional long short-term memory (LSTM) [49], to predict the transcriptional direction of enhancers, both being recognized for their efficacy in enhancer sequence prediction. For details on the code and usage of these two deep learning models, please refer to https://github.com/minxueric/ismb2017_lstm. In our analysis, we used the 2-kb enhancer region corresponding to the eRNA as the input sequence for the model. We treated 4832 unidirectional transcribed enhancers as negative samples and 12,883 bidirectional transcribed enhancers as positive samples. To split the dataset, we used 85% of the data for training, 5% for model selection as a validation set, and the remaining 10% for testing the model. We evaluated the performance of the two models on the test data by calculating the area under a receiver operating characteristic (ROC) curve (AUC) value, the ROC curves were plotted using the plotROC [50] package (version 1.3) in R.

### Transposon analysis

To annotate transposons in the pig genome, we used the RepeatMasker [51, 52] software (version 4.1.2-p1) and the Repbase-20181026 library (RepeatMasker -parallel 15 -species pig -html -e rmblast -s -a -gff -dir pig_repeat susScr11.fa). We categorized enhancers into two groups: transcribed enhancers (TEn and non-transcribed enhancers (non-TEn). Specifically, enhancers that intersect with the center points of eRNAs were referred to as TEn, while enhancers without intersection with the center points of eRNAs were termed non-TEn. We then performed a comparative analysis of transposon insertions within TEn and non-TEn using the "intersect" command of the BEDTools (v2.31.0) software. First, we examined the differences in transposon insertion frequencies between TEn and non-TEn using a two-tailed Fisher’s exact test. Furthermore, we calculated the base composition of different classes and families of transposons within TEn and non-TEn using R, and all results were visualized using the ggplot2 package. To assess the differential enrichment of transposon families in TEn and non-TEn, we performed a permutation test by simulating elements 1000 times within the genomic background. The resulting p-values were corrected by the false discovery rate (FDR) method. The enrichment results were then visualized using the pheatmap package (version 1.0.10) in R.

### Identification of tissue-specific eRNAs and genes

To identify tissue-specific eRNAs and genes, we retrieved and analyzed strand-specific RNA-seq data from the heart, liver, and spleen tissues of both Duroc and ES pigs. It is essential to emphasize that these pigs were the same individuals than those used for enhancer identification in the current study. Each tissue type was represented by two biological replicates. The quality control and alignment methods for the transcriptome data were consistent with those mentioned earlier. Gene counts were quantified using FeatureCounts (version 2.0.2) [53] on the bam files of each sample. We calculated the gene expression level (TPM) based on the raw count expression matrix, taking gene length and library depth into account using an R script. In addition, we evaluated the expression levels (RPM) of detectable eRNAs in each tissue using the Seqmonk software (Babraham Institute), which is consistent with the previously described quantification methods. Finally, we identified tissue-specific eRNAs and genes for each breed by analyzing the expression profiles across five tissues: muscle, fat, heart, liver, and spleen. Specifically, it is important to note that the tissue specificity of eRNAs was calculated separately for biological replicates 1 and 2. This analysis was conducted using the tissue specificity index (TSI) [54], which is calculated as follows:

$${\text{TSI}} = \frac{{\mathop \sum \nolimits_{{{\text{i}} = {\text{i}}}}^{{\text{n}}} \left( {1 - x_{{\text{i}}} } \right)}}{{{\text{N}} - 1}},$$

where ${\text{N}}$ is the number of tissues and ${x}_{{\text{i}}}$ is the expression of the eRNA or gene $x$ in tissue ${\text{i}}$. Genes or eRNAs with a TSI greater than 0.8 in both biological replicates of the specific tissue in the same breed were deemed to be tissue-specifically expressed in that particular breed.

### Enrichment analysis of tissue-specific eRNAs

In this study, we conducted a hypergeometric test to assess the enrichment of tissue-specific expressed eRNAs within super-enhancers in eastern and western pig muscle and adipose tissues. This analysis used key parameters including the total number of detectable eRNAs in each tissue (T), the number of detectable eRNAs within super-enhancers (M), the number of tissue-specific detectable eRNAs (t), and the number of tissue-specific detectable eRNAs within super-enhancers (m). In addition, we performed a hypergeometric test to assess the enrichment of tissue-specific expressed genes within ± 1 Mb regions of tissue-specific eRNAs. In this analysis, X denotes the total number of detectable genes in the tissue, Y represents the count of detectable genes within ± 1 Mb of eRNAs, x indicates the number of tissue-specific expressed genes, and y represents the count of tissue-specific expressed genes within ± 1 Mb of eRNAs.

To explore the potential involvement of TF in regulating tissue-specific eRNAs, we performed motif enrichment analysis using the HOMER [55] software on H3K27ac histone peaks within the regions of tissue-specific eRNAs. We focused on the top 20 enriched TF motifs within tissue-specific eRNAs and retained only the TF that showed significant enrichment (Q-value < 0.01) and were expressed in the corresponding tissue. To visualize the enrichment levels, we used the ggplot2 package for data visualization.

To investigate the contribution of tissue-specific eRNAs to the heritability of tissue-related biological traits, we obtained datasets from pigQTLdb, which contained GWAS hits and QTL regions associated with various pig traits. These traits were classified into five major classes: Meat and Carcass, Health, Production, Reproduction, and Exterior. After downloading the datasets, we processed and extracted the location information for GWAS hits and QTL regions associated with each trait class. Next, we performed permutation tests by simulating tissue-specific eRNA elements within a genomic background, repeating the process 1000 times (see Additional file 1 Supplementary code). This allowed us to assess the enrichment of GWAS hits and QTL regions associated with different trait classes in tissue-specific eRNAs. It is important to note that we expanded the GWAS hits by ± 20 kb to account for the potential influence of linkage disequilibrium (LD) among SNPs.

To investigate the contribution of pig tissue-specific eRNAs in elucidating the genetic heritability of human diseases, we obtained the susScr11ToHg19.over.chain file from the UCSC database and employed the liftOver tool (https://hgdownload.soe.ucsc.edu/admin/exe/linux.x86_64/liftOver) [56] to convert pig tissue-specific eRNAs into homologous sequence regions in humans. specifying a length of 6 kb and a minimum match threshold of 0.5. Subsequently, we downloaded GWAS summary statistics for 64 traits from Hook’s study [37]. Using the LDSC method [57], we dissected the heritability of these 64 complex traits in humans based on the pig-driven human tissue-specific expression of eRNAs. Finally, we combined and analyzed the partitioned heritability contributions for all traits and visualized the results in R.

### Construction of an eRNA regulatory network

To construct directed eGRN based on tissue-specific eRNAs, we used a unique approach that involved identifying the upstream TF and downstream target genes of eRNAs. For the identification of upstream TF, we used a combination of eRNA sequence motif scanning and co-expression analysis between TF and eRNA expression levels. Initially, we obtained the custom motif matrices files for TF, which contain motif sequences (http://homer.ucsd.edu/homer/custom.motifs). We then extracted the DNA sequence corresponding to the enhancer region of each eRNA from the susScr11.fa file using the “getfasta” command provided by BEDTools (v2.31.0). Motif scanning was conducted using the scanMotifGenomeWide.pl command (scanMotifGenomeWide.pl custom.motifs element.fa -bed) within the HOMER software. TF with motif sequences that could be detected on the DNA sequence and were expressed in the tissue were considered as potential upstream regulators of eRNAs. The confirmation of these potential upstream TF as true regulators of the eRNAs was based on their significant correlation with the regulation of eRNA expression in 20 samples (Rs > 0.5, FDR < 0.05). The correlation analysis was performed using the ‘spearman’ method from the psych package (version 2.2.5) in R, with adjustment for multiple testing using the FDR method. For the identification of target genes of eRNAs, we employed a similar strategy of co-expression analysis between eRNAs and genes in 20 samples. The expression correlation calculation method was consistent with the previous approach, with a threshold set at Rs > 0.3 and FDR < 0.05. In addition, the target genes had to be located within a 1 Mb range of the eRNA. To further filter the target genes of eRNAs, we leveraged the conserved nature of the topologically associated domain (TAD) structure in the 3D genome across species [58]. We used TAD regions derived from 2-week-old large white pig muscle tissue and required that both the eRNA and its target genes be located within the same TAD region. Lastly, to enhance the elucidation of the regulatory mechanisms that underlie the traits, we integrated population-based GWAS data into our analysis. Specifically, we extracted GWAS hits associated with fat and muscle traits from pigQTLdb (see Additional file 2: Table S1). To account for the LD effect of SNPs, we specifically screened for eRNAs that intersected with the GWAS hits within a ± 20 kb region. This approach allowed us to identify eRNAs with potential regulatory effects on the corresponding traits. Subsequently, we constructed separate eGRN using the eRNAs associated with muscle and fat traits to uncover the genetic regulatory mechanisms underlying these traits. The resulting eGRN were visualized using the Cytoscape [59] software. To further refine the eGRN in fat tissue, we performed capture STARR-Seq experiments in mouse 3T3 cells, in order to identify eRNA SNPs that affect enhancer activity. In the end, we removed eRNAs without enhancer-modulating SNPs in the final eGRN of the fat tissue.

### Cell culture

Mouse 3T3-L1 fibroblasts (ATCC) were initially cultured in DMEM supplemented with 10% calf serum (B7446, Sigma-Aldrich) to promote cell proliferation. During the induction of cell differentiation, a transition from calf serum to 10% fetal bovine serum (FBS) (10091148, Gibco) was carried out upon the initial phase of cell contact inhibition. The aim of this substitution was to enhance the initiation of lipid droplet formation effectively. Two days after replacement of the FBS, the induction of differentiation was accomplished by incubating the cells in differentiation medium A (DMEM containing 0.5 mM IBMX (I5879, Sigma), 1 μM DEX (D1756, Sigma-Aldrich), and 10 μg/mL insulin (HY-P0035, MedChemExpress), and 10% FBS) for 2 days, followed by differentiation medium B (DMEM containing 10 μg/mL insulin and 10% FBS) for subsequent days with a medium change performed every two days to achieve full differentiation into mature adipocytes (Day 8).

### SNP screening for STARR-seq

To identify regulatory SNPs in the fat-related eGRN that regulate differential fat deposition abilities between eastern and western pigs, we established screening criteria for SNPs that were assessed using STARR-seq for regulatory activity. Initially, we obtained genome-wide SNP data from the PigVar database [60] for eastern and western pig populations. We then overlapped this data with eRNAs to identify the SNP locations within eRNAs. To ensure the detection of SNPs within eRNAs in both eastern and western pig populations collected by our research group, and to assess their potential influence on the differential fat phenotypes between the two groups, we focused on SNPs that showed allele frequency differences between the eastern and western pig populations collected by our team, with a minor allele frequency (MAF) difference exceeding 0.3, and ensuring that the MAF in the whole genome mixed pool of eight eastern and eight western pigs was higher than 0.05. SNPs that met the selection criteria were chosen as candidate SNPs for the STARR-seq assay, allowing for the detection of their regulatory activity on DNA elements. The protocol for the study involving the 16 pigs obtained approval from Huazhong Agricultural University (Protocol code: SYXK(e)2020-0084) and the Institutional Animal Care and Use Committee.

### Identification of regulatory SNPs by STARR-Seq

Candidate SNPs with regulatory functions were identified using a previously reported STARR-Seq strategy [35, 61]. Initially, custom primers for the 22 enhancer elements containing candidate SNPs were designed and synthesized by Sangon Biotech Co., Ltd (Shanghai, China) (see Additional file 3: Table S2). To introduce SNP polymorphisms into the screening process, DNA samples extracted from various tissues of eight eastern and eight western pigs were pooled in equal amounts and used as PCR templates. The PCR amplification parameters were 25 cycles consisting of 94 °C for 5 min, 95 °C for 30 s, 55 °C for 30 s, 72 °C for 1 min, and a final extension at 72 °C for 7 min. The reaction mixture, with a total volume of 50 μL, contained 1 μg of substrate and 0.4 μM of primers. The amplified products were purified and sonicated to achieve sizes ranging from 300 to 400 bp. These sonicated fragments were ligated into the hSTARR-seq_ORI vector (#99296, addgene). The recombinant vectors were transformed into *E. coli* DH10B cells (Life Technologies, Eragny, France) by a Gene Pulser Xcell Electroporation System (Bio-Rad Laboratories, Richmond, CA, USA) to produce the input plasmid library, which then was transfected into 3T3-L1 cells by lipofectamine (jetPRIME, Polyplus 101000046). The STARR-Seq input NGS library was derived from the input plasmid library, whereas the output NGS library was generated from transcripts produced by the input plasmids. To be clear, the input NGS library was created by directly amplifying SNP-containing inserts from the plasmid DNA used for cell transfection, thereby serving as a reference for the initial representation of SNP alleles in the starting plasmid pool. In contrast, the output NGS library assesses the abundance of self-transcribed mRNA originating from insert fragments within the transfected plasmid pool. The experimental details of STARR-Seq are described in previous publications [35, 62]. Briefly, to prepare the input NGS library, the input library was prepared using 200 ng of plasmid template, divided into four reactions of 50 μL each. PCR amplification was carried out with the specified primers containing Illumina adaptors [forward: 5ʹ-AATGATACGGCGACCACCGAGATCTACAC-index-ACACTCTTTCCCTACACGACGCTCTTCCGATCT-3ʹ; reverse: 5ʹ-CAAGCAGAAGACGGCATACGAGAT-index-GTGACTGGAGTTCAGACGTG-3ʹ] and the following conditions: 95 °C for 3 min, 98 °C for 20 s, 65 °C for 15 s, 72 °C for 30 s (go to step 2 for 15 cycles, 72 °C for 2 min). To prepare the output NGS library, the transfected cells were harvested after 24 h. Total RNAs were extracted, and mRNAs were enriched by oligodT and reverse transcribed using a plasmid specific primer [CAAACTCATCAATGTATCTTATCATG]. Following reverse transcription, the cDNA products were purified and amplified with primers containing Illumina adaptors to construct the output NGS libraries [forward: 5ʹ-AATGATACGGCGACCACCGAGATCTACAC-index-ACACTCTTTCCCTACACGACGCTCTTCCGATCT-3ʹ; reverse: 5ʹ-CAAGCAGAAGACGGCATACGAGAT-index-GTGACTGGAGTTCAGACGTG-3ʹ]. The PCR amplification was performed using the KAPA enzyme (KK2602, Roche) with the following cycling conditions: 95 °C for 3 min, 98 °C for 20 s, 65 °C for 15 s, 72 °C for 30 s (go to step 2 for 18 cycles, 72 °C for 2 min). Both the input NGS library and the output NGS libraries were sequenced using the PE150 strategy on the NovaSeq 6000 platform. In this study, we obtained four output NGS libraries (the RNAs from each biological replicate generated two output libraries) and one input NGS library, the output libraries yielded a range of 2.8 to 3.4 million reads each, with the input library containing 8.5 million reads. After performing quality control and removing PCR duplicates, the reads from the two biological replicates were highly correlated (see Additional file 4: Figure S1a), Therefore, we merged the two replicates for further analysis to increase statistical power for calling regulatory SNPs. When calculating the effect size of SNPs, only SNPs with a coverage exceeding 20 in both input and output libraries were considered. The size of the effect of each SNP was determined by calculating the fold change in allele ratios (output/input). Significance of the differences in allele ratios was assessed using a two-tailed Fisher's exact test. The raw sequence data of STARR-seq used in this paper have been submitted to the Genome Sequence Archive (GSA; https://ngdc.cncb.ac.cn/) under accession number CRA011292.

### Gene function validation by siRNA

The siRNAs targeting the *RB1*, *SLC27A6*, *RGMA*, *APLF*, *UBTD2* genes, and the negative control were custom-synthesized by Ribo Biological Co., Ltd (Guangzhou, China) (see Additional file 5: Table S3). 3T3-L1 cells were seeded in a six-well culture plate and transfected at 80% confluency. The medium was refreshed 4 to 6 h after the transfection process. To ensure continuous suppression of the target gene expression at a low level, a subsequent knockdown was conducted on the fourth day of the induced differentiation process, with a repeat transfection of siRNA. Then, RNA was extracted using the TriZol reagent, purified by chloroform extraction, precipitated with isopropanol, and dissolved in RNase-free water. Subsequently, 1 μg of RNA was used for cDNA synthesis through reverse transcription in a 20-μL reaction. ChamQ Universal SYBR qPCR Master Mix (Q711 Vazyme) was used for RT-qPCR, 1 μL of cDNA as templates and 0.2 μM primers. The PCR conditions included an initial denaturation at 95 °C for 30 s, followed by 40 cycles of 95 °C for 10 s, 60 °C for 30 s, and a final step of 95 °C for 15 s, 60 °C for 60 s, and 95 °C for 15 s. The degree of differentiation was assessed by fluorescence microscopy and reverse transcription (RT)-qPCR analyses. Finally, we used the GraphPad Prism 5 software to perform a t-test to compare the expression levels of target genes and marker genes with those of the negative control, and then visualized the expression levels accordingly.

### Oil red O staining

When 3T3-L1 cells were fully differentiated into adipocytes (after 8 days), cells were fixed with a 4% formalin solution (BL539A, biosharp) for 15 min at room temperature. The fixed cells were then washed three times with phosphate buffered saline (PBS) (C10010500BT, Gibco) for 1 min each and stained with Oil Red O (G1260, Solarbio) at 37 °C for 1 h. Stained cells were washed twice with 60% isopropanol (1.17029.023, GHTECH) for 1 min each and then washed with PBS, 4 to 5 times for 1 min each. Formation of lipid droplets was observed under a microscope.

## Results

### Identification and feature analysis of eRNAs in muscle and fat tissues

**Fig. 1:**
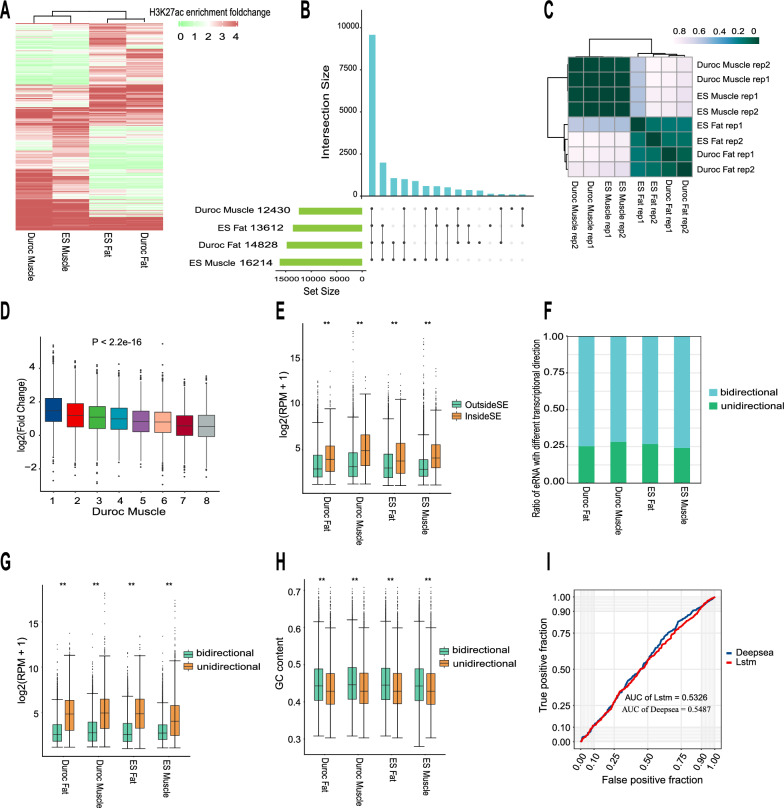
Identification and regulatory characteristics of eRNAs. **a** A heatmap revealed enhancer intensity dynamics in muscle and adipose tissues between Enshi Black (ES) and Duroc pigs. Enhancer intensity was calculated by normalized H3K27ac ChIP-seq signals. **b** Overlap of detectable eRNAs between different tissues and breeds. The bar chart in light green displays the number of detectable eRNAs for each tissue, the blue diagram illustrates the degree of overlap between detected eRNAs across different tissues and breeds. **c** Heatmap displaying the calculated Euclidean distances between samples based on the variance-stabilizing transformation of the eRNA expression matrix. **d** Pearson correlation between eRNA expression and enhancer activity in Duroc muscle tissue. **e** Comparison of eRNA expression levels within and outside of super-enhancers. Statistical significance was assessed using the two-sided unpaired Wilcoxon test (**P < 2.2e−16). **f** Proportion of detectable unidirectional- and bidirectional-transcribed eRNA per tissue. **g** Expression levels of unidirectional- and bidirectional-transcribed eRNAs in each tissue. Statistical significance was assessed using the two-sided unpaired Wilcoxon test (**P < 2.2e−16). **h** GC content of unidirectional- and bidirectional-transcribed eRNA sequences in each tissue. Statistical significance was assessed using the Student’s t-test (**P < 2.2e−16). **i** Deep learning model distinguishes transcriptional directions of eRNAs

To identify eRNAs on a genome-wide scale in muscle and fat tissues from both ES and Duroc pigs, first we identified enhancers defined based on H3K27ac signal intensity [36], in line with the recognized definition that eRNAs are transcribed from enhancer regions. In total, we identified 69,881 enhancers in muscle and fat tissues from ES and Duroc pigs. Among them, 31,336, 27,148, 51,898, and 36,635 enhancers were identified in Duroc muscle, Duroc fat, ES muscle, and ES fat, respectively (see Additional file 6: Table S4). Samples were clustered based on H3K27ac signal intensity, which showed that enhancer activity was more conserved between breeds than between tissues (Fig. 1a). Next, based on the coordinates of these enhancers, we used RNA-seq data from the same individuals for which H3K27ac data were used to quantify the expression levels of eRNAs in each respective tissue, referring to the identification methodology used in a previous eRNA study [29]. Using RNA-seq data, the expression level of eRNAs was quantified with the SeqMonk software, which is referred to as the Nancy method [30] (see Additional file 7: Figure S2a). Finally, we identified 17,715 eRNAs, of which 12,430, 14,828, 16,214, and 13,612 were detected in Duroc muscle, Duroc fat, ES muscle, and ES fat, respectively (see Additional file 8: Table S5). eRNA expression clustering revealed high reproducibility between biological replicates and a higher expression similarity between breeds than between tissues (Fig. 1c). Although eRNA expression was found across all pig breeds and tissues, a small number of the eRNAs were expressed in a tissue/breed specific pattern (Fig. 1b and see Additional file 9: Figure S3). In each tissue, the expression of eRNA is positively correlated with H3K27ac enhancer activity (P < 2.2e−16), which is consistent with prior investigations in this field (Fig. 1d and see Additional file 10: Figure S4a–c). We also identified SE in muscle and fat tissues from ES and Duroc pigs (see Additional file 11: Figure S5a–d). The significantly higher expression of eRNAs within than outside SE (P < 2.2e−16) (Fig. 1e), underscored the potential of eRNAs to regulate key genes that define cell identities [63, 64].

eRNA transcription was previously known to be bidirectional, but subsequent studies have shown that not all eRNAs are bidirectionally transcribed [65, 66]. Consistently, we found that only about 3/4 of the eRNAs in each tissue were bidirectionally transcribed (Fig. 1f). Compared to unidirectionally transcribed eRNAs, the bidirectionally transcribed eRNAs have lower expression (Fig. 1g) and higher GC levels (Fig. 1h). Furthermore, the adjacent gene sets associated with both unidirectional and bidirectional transcribed eRNAs demonstrate enrichment in distinct biological processes (see Additional file 7: Figure S2b). In spite of a modest but significant difference in GC content for these two eRNA classes, distinguishing enhancers with distinct transcription directions posed a challenge when relying on higher-level sequence features. Using deep learning methods, including DeepSEA [48] and LSTM [49], we observed limited discriminatory power, with AUC values of 0.54 and 0.53, respectively (Fig. 1i). To the best of our knowledge, this compilation of bidirectional and unidirectional eRNAs represents the first comprehensive eRNA expression profile documented in pig tissues.

### The long terminal repeat (LTR) retrotransposon family might promote enhancer transcription

**Fig. 2:**
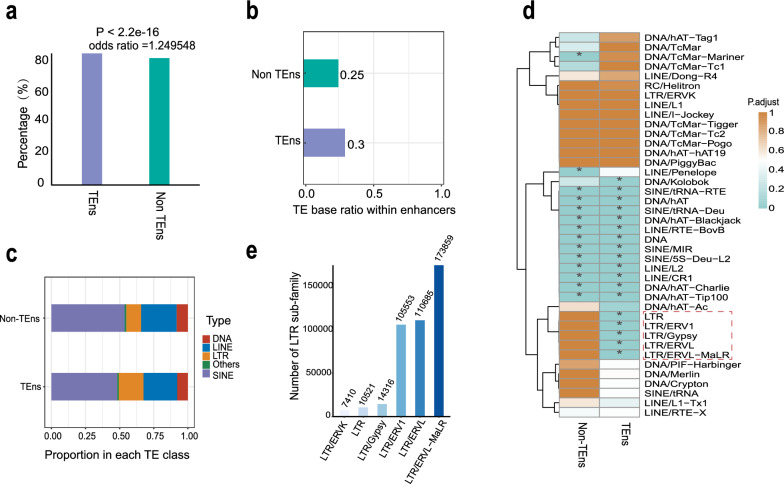
The effect of transposons on enhancer transcription. **a** Percentages of transposon insertion in transcribed enhancers (TEn) and non-transcribed enhancers (non-TEn). Statistical significance was assessed using Fisher’s exact test. **b** Proportion of transposon bases within TEn and non-TEn. **c** Proportion of enhancer occupancy by TE classes. **d** Enrichment analysis of transposon family in TEn and non-TEn. Statistical significance was determined using a permutation test with genomic background simulation of elements performed 1000 times and adjusted false discovery rate (FDR) (*P < 0.05). **e** Number of long terminal repeats (LTR) retrotransposon families annotated in the pig genome

In mammals, transposon sequences account for about half of the entire genome [67]. They play a significant role in the generation of numerous cis-regulatory elements by incorporating sequence features that are rich in information into the genome, including TF binding motifs [68, 69]. In our study, about 81% of the identified enhancers marked by H3K27ac had transposon insertions, which implies that transposons have an important role in the formation of enhancers. However, whether certain types of transposons preferentially contribute to the formation of eRNA loci is unexplored. Following this idea, we classified the enhancers into two categories: transcribed enhancers (TEn) and non-transcribed enhancers (non-TEn). We found that non-TEn (79%) had subtle but significantly lower frequencies of transposon insertion events than TEn (83%) (Fig. 2a; *P* < 2.2e−16). The percentage of transposon bases in TEn was also higher than in non-TEn (Fig. 2b), and this difference was mainly due to an increased presence of the long terminal repeat (LTR) retrotransposon family in TEn, compared to short (SINE), and long (LINE) interspersed sequences, DNA repeats, or other types of repeats (Fig. 2c), which indicates a more prominent role of retroviral LTR in the transcription of enhancers. To further study the effect of the LTR retrotransposon family on the transcription of enhancers, we performed enrichment analysis of different transposon families in enhancers. All LTR families were significantly and specifically enriched in TEn compared to non-TEn except for the LTR/ERVK family (Fig. 2d). The limited enrichment of this specific LTR family in the TEn may be attributed to its infrequent presence within the genome, which subsequently reduces the statistical robustness of the enrichment analysis (Fig. 2e). In summary, we infer that the insertion of LTR sequences might play an important role in eRNA transcription.

### Tissue-specific eRNAs exhibit robust regulatory potential in tissue-specific-biological processes

Next, we explored the tissue specificity of eRNAs, as the tissue-specific regulatory landscape is thought to have a substantial impact on trait and disease etiology [70–72]. Our hypothesis was that eRNAs that are specifically expressed in pig muscle and fat tissues are closely related to pork production traits. To this end, we downloaded additional RNA-seq data from heart, liver and spleen tissues of ES and Duroc pigs (GEO Data repository GSE143288). Then, we analyzed the expression level of the 17,715 eRNAs in five tissues across the two breeds (see Additional file 12: Table S6). The heatmap clustering analysis revealed a striking consistency in eRNA expression patterns across these two breeds within the same tissue, quantified in reads per million (RPM). While most biological replicates displayed cohesive clustering, it is noteworthy that, in one case, a replicate of skeletal muscle tissue from the ES breed clustered with ES heart tissue. This deviation could be ascribed to the underlying physiological and functional similarities between the skeletal and cardiac muscles [73] (see Additional file 9: Figure S3). Interestingly, this similarity in eRNA expression within tissues transcended breed variations. Conversely, when examining a particular breed across different tissues, the eRNA expression patterns displayed more divergence. This analysis underscores the presence of tissue-specific eRNAs, which implies that they play vital roles in orchestrating tissue-specific regulatory processes.

**Fig. 3:**
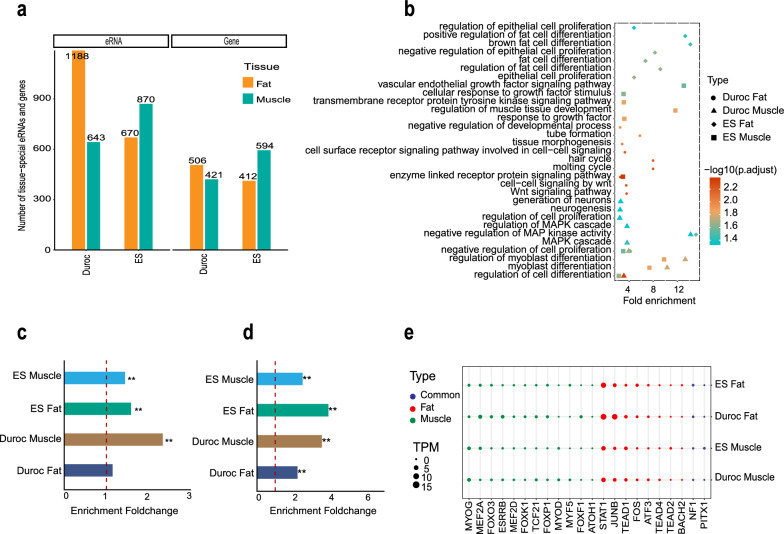
Analysis of the biological functions and the potential regulatory mechanisms of tissue-specific eRNAs. **a** The number of tissue-specific eRNAs and genes in each tissue between Enshi Black (ES) and Duroc pigs. **b** Gene ontology (GO) analysis reveals biological process pathways relevant to eRNAs expressed in a tissue- and breed-specific manner. GO enrichment analysis was performed based on the neighboring genes of eRNAs. **c** Tissue-specific genes were significantly enriched within ± 1 Mb distance of tissue-specific eRNAs. Statistical significance was assessed using the hypergeometric test (**P < 0.01). **d** Enrichment analysis of tissue-specific eRNAs in super-enhancers (SE). Statistical significance was assessed using the hypergeometric test (**P < 0.01). **e** Motif enrichment analysis of tissue-specific eRNAs. Red dots represent transcription factors (TF) with a DNA binding motif that were significantly enriched in adipose-specific eRNAs; green dots represent those that were significantly enriched in muscle-specific eRNAs; dark blue dots represent those that were significantly enriched in both fat and muscle-specific eRNAs. The size of the dots represents the expression level of corresponding TF

Next, we used the tissue specificity index (TSI) algorithm to identify tissue-specific eRNAs in each tissue of each breed (see “Methods” for details). In total, we identified 643, 1188, 870, and 670 eRNAs specifically expressed in Duroc muscle, Duroc fat, ES muscle, and ES fat tissues, respectively (Fig. 3a and see Additional file 13: Table S7). The genes that were localized nearest to these tissue-specific eRNAs were significantly enriched in biological pathways that are involved in the differentiation and development of the corresponding tissues (Fig. 3b). Although the neighboring genes of Duroc fat-specific eRNAs did not exhibit direct enrichment in pathways specifically associated with fat deposition, they displayed enrichment in pathways relevant to adipogenesis, such as the Wnt signaling pathway [74, 75]. However, the neighboring genes of non-tissue-specific eRNAs exhibited a remarkable pattern of enrichment in essential biological pathways that are shared across diverse tissues (see Additional file 14: Figure S6). These GO analyses suggested that tissue-specific eRNAs play an important role in regulating biological processes that are relevant to tissue identity. With the exception of Duroc adipose tissue (*P* = 0.056), we found that tissue-specific eRNAs were significantly enriched within SE of the corresponding tissues (Fig. 3c). Furthermore, we identified 421, 506, 594 and 412 genes that were specifically expressed in Duroc muscle, Duroc fat, ES muscle and ES fat tissues, respectively (Fig. 3a and see Additional file 13: Table S7). In alignment with the adjacent genes of tissue-specific eRNAs, tissue-specific genes were also significantly enriched in biological pathways that are relevant to tissue identity, except for Duroc fat (see Additional file 15: Figure S7). The functional relevance of the tissue-specific eRNAs and genes led us to speculate that tissue-specific genes are primarily regulated by tissue-specific eRNAs. To explore the potential regulatory relationship between tissue-specific eRNAs and tissue-specific genes, we conducted an enrichment analysis comparing tissue-specific genes to tissue-specific eRNAs within a ± 1 Mb region, which is a commonly employed criterion for assigning putative target genes to eRNAs [27, 31]. Our findings revealed a significant enrichment of tissue-specific gene locations in tissue-specific eRNAs within ± 1 Mb, in the same tissue (Fig. 3d). This enrichment implies that tissue-specific eRNAs may have robust regulatory activities on the expression of tissue-specific genes. To investigate which TF might be involved in the regulation of tissue-specific eRNAs, we performed TF binding motif enrichment analysis of tissue-specific eRNAs. As expected, the top enriched motifs in tissue-specific eRNAs are DNA binding motifs of master TF that are closely related to tissue development. For example, MYOG, MYOD, MYF5, MEF2A, and MEDF2D were significantly enriched in muscle-specific eRNAs, while ATF3 [76, 77], BACH1 [78], and STAT1 [79] were significantly enriched in fat-specific eRNAs (Fig. 3e; Q-value < 0.01). Therefore, tissue-specific eRNAs are likely to function downstream of these master TF to establish and maintain tissue identity. Accordingly, DNA variants that dysregulate these eRNAs might significantly contribute to the genetic components underlying pork economic traits relevant to muscle and fat.

### Genetic contribution of tissue-specific eRNAs to tissue-relevant traits

**Fig. 4:**
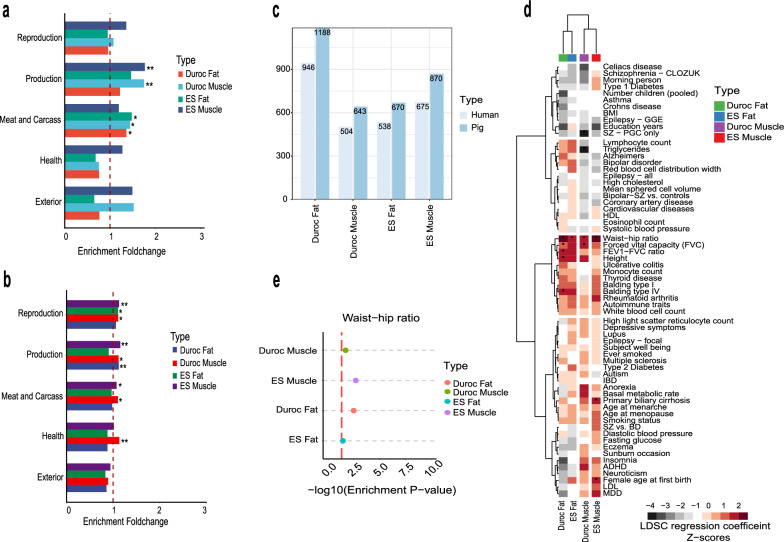
Contribution of tissue-specific eRNAs for the genetic basis of pig economic traits. **a** Enrichment of genome-wide association study (GWAS) signals of five major pig economic traits in tissue-specific eRNAs. A permutation test was performed with genomic background simulation of eRNA elements conducted 1000 times and a false discovery rate (FDR) adjusted for statistical significance (*P < 0.05; **P < 0.01). **b** Enrichment of tissue-specific eRNAs in trait-related quantitative trait loci (QTL) regions. Statistical significance was determined using a permutation test with an adjusted FDR (*P < 0.05; **P < 0.01). **c** Number of pig tissue-specific eRNAs and their homologs in the human genome. **d** A heatmap displaying stratified linkage disequilibrium score regression (S-LDSC) heritability enrichment of 64 human GWAS traits in human homologs of pig tissue-specific eRNAs. Data are hierarchically clustered by GWAS and tissues. * represents significant enrichment (P < 0.05). **e** A dot plot displaying the statistical significance of S-LDSC enrichment for waist-hip ratio. A red dashed line is marked at −log10(0.05)

Numerous post-GWAS studies have dissected the heritability of complex traits by fine-mapping DNA variants in the non-coding regions of genomes [80, 81]. Thus, we postulate that tissue-specific eRNAs might provide a unique resource to functionally interpret GWAS signals that lie outside of coding regions, and thereby pinpointing novel causal regulatory elements and genes. To explore how muscle- and fat-specific eRNAs might shed light on the genetic basis of muscle- and fat-relevant pork production traits, we first collected all GWAS hits and QTL associated with different porcine traits from the pig QTLdb [82]. These traits are categorized into five major groups (Meat and Carcass, Health, Production, Reproduction, Exterior) in the pigQTLdb database. We observed a significant and specific enrichment of muscle- and adipose-specific eRNAs within 20-kb linkage regions in the GWAS signals related to meat and carcass traits, as well as to production traits (Fig. 4a). These two groups of traits are largely affected by muscle and fat development. In the enrichment analysis using QTL regions, muscle- and fat-specific expression eRNAs were not only enriched in muscle and fat development-related traits (Fig. 4b), but also in reproductive and health traits. This lack of enrichment specificity might be due to the fact that the pig QTL regions were generally much longer than the GWAS regions.

To further explore the biological importance of muscle- and fat-specific eRNAs, we took advantage of the human GWAS, which usually provide sharper GWAS signals, and evaluated how the human orthologs of these tissue-specific eRNAs contribute to various human traits. The tissue-specific eRNAs exhibit a high level of sequence homology between pigs and humans, and the majority of them could be successfully lifted over to the human genome (Fig. 4c). In the comparison between our results and the summary statistics data from 64 GWAS for human traits collected by Hook and McCallion [37], the stratified linkage disequilibrium score regression (S-LDSC) analysis revealed that the human orthologs of pig muscle- and fat-specific eRNAs were significantly enriched in waist-hip ratio GWAS associated loci (Fig. 4d, e and see Additional file 16: Table S8). This finding is consistent with the notion that the regulation of waist-hip ratio involves coordinated mechanisms that govern muscle growth and fat deposition. Thus, we provided evidence that pig muscle- and fat-specific eRNAs might constitute highly conserved transcriptional regulatory networks that underlie crucial muscle- and fat-relevant phenotypes across pigs and humans.

### Construction of eRNA-mediated gene regulatory networks underlying muscle- and fat-relevant traits

**Fig. 5:**
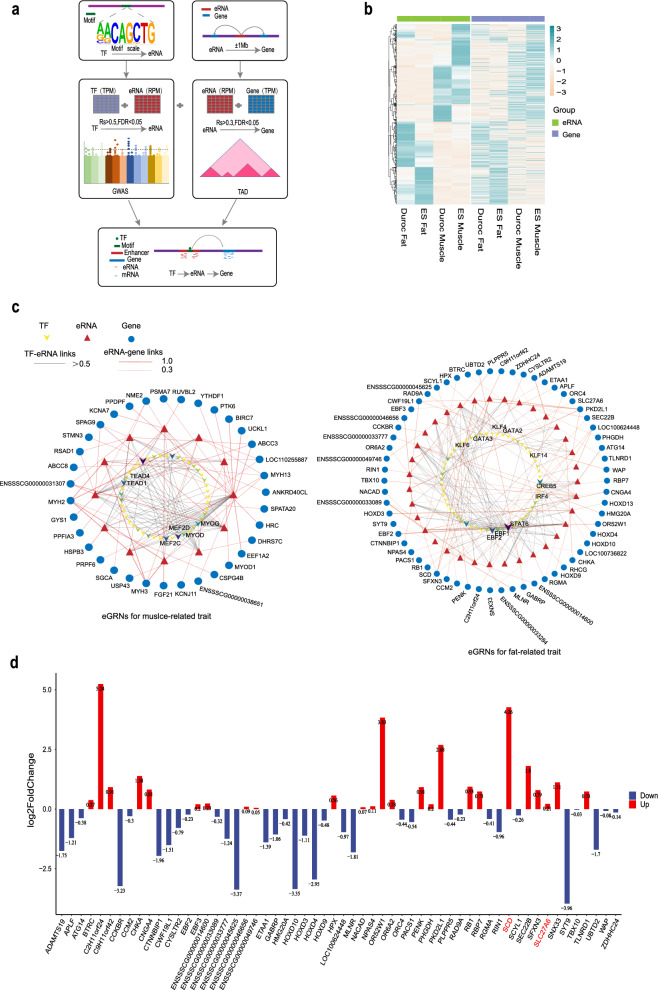
Construction of potential trait-affecting eRNA-mediated gene regulatory networks (eGRN) by integrating multi-omics data. **a** Schematic diagram of the eGRN construction method. Motif scanning and expression correlation between transcription factors (TF) and eRNA expression levels were used to predict upstream TF of eRNAs. The distance between eRNAs and genes and the expression correlation between their expression levels were used to predict downstream target genes of eRNAs, topologically associated domain (TAD) structure was further used to filter out eRNAs and genes that were not in a same TAD. Genome-wide association study (GWAS) data were used to further filter for trait-associated hub eRNAs that harbor DNA variants that are associated with pig economic traits. **b** Heat maps showing the globally correlated changes of the expression of eRNAs and target genes. **c** eGRN for muscle- and fat-related traits. The color of a TF represents the number of its downstream regulatory eRNAs, where a darker color indicates a larger number of regulatory eRNAs. **d** Differential expression of eRNA target genes between ES and Duroc pigs (ES vs Duroc) in fat-related eGRN

To pinpoint tissue-specific eRNAs that potentially influence meat-relevant traits, we present a novel approach to construct gene regulatory networks. This innovative method enables the identification of eRNAs, which harbor DNA variants that are likely to contribute to phenotypic variation. Compared to previous co-expression-based gene regulatory networks (GRN) that link genes with undirected edges [83], our networks (referred to as eGRN) are eRNA-centric, have directed edges and leverage information from population genetics studies. Our methodology involves several key steps. First, we used co-expression analysis and motif scanning to associate upstream TF with tissue-specific eRNAs. Next, we used a combination of co-expression patterns, a 1-Mb distance cutoff, and topologically associated domain (TAD) boundaries to link these eRNAs to potential downstream target genes. Subsequently, we leveraged pig GWAS data to keep only the gene regulatory networks mediated by eRNAs that harbor DNA variants exhibiting potential associations with meat-relevant traits (Fig. 5a, and see “Methods”).

Based on our approach, 61% of the tissue-specific expression eRNAs were assigned to at least one target gene and most of the eRNAs were assigned to no more than five (1680/2751) (see Additional file 17: Figure S8A). eRNAs and their target genes showed notable and coordinated expression changes, which suggest a robust and synergistic relationship between them (Fig. 5b). Gene ontology enrichment analyses revealed that although the target genes of Duroc fat- and ES muscle-specific eRNAs were not enriched in tissue-related pathways, those of Duroc muscle- and ES fat-specific eRNAs were both significantly enriched in tissue-related biological pathways (see Additional file 17: Figure S8b). This pattern is reminiscent of the selection for muscle mass and fat deposition in Duroc and ES during pig breeding practice, respectively, indicating distinct regulatory roles of the tissue-specific eRNAs that underlie selection during the domestication and breed development of pigs. The eGRN of muscle and fat tissues encompass 1023 and 711 regulatory relationships, respectively, involving upstream TF binding to eRNA-transcribing enhancers for the regulation of downstream target genes (Fig. 5c and see Additional file 18: Table S9). Excitingly, we found that the upstream TF of these eRNAs include many known master regulators for tissue development, such as MYOG, MYOD, MYF5, MEF2A, and MEDF2D in the muscle eGRN, and CREB5, IRF4, EBF1, EBF2, KLF6, KLF4, GATA2, GATA3, KLF14, and STAT6 in the fat eGRN [84, 85]. In addition, the target genes of the eGRN also include genes that have been reported to regulate tissue development, such as *MYOD1*, *MYH1*, *MYH2* in the muscle eGRN, as well as *SCD* and *SLC27A6* in the fat eGRN. Interestingly, the expression level of *MYOD1, MYH1, MYH2* was higher in Duroc muscle than in ES muscle, while the expression level of *SCD* and *SLC27A6* was higher in ES fat than in Duroc fat (Fig. 5d and see Additional file 17: Figure S8c). This difference in gene expression is consistent with differences in lean mass ratio and fat deposition between Duroc and ES pigs. Taken together, these results provide compelling support for the reliability of our eGRN in capturing essential transcriptional regulatory units that underlie relevant traits.

### Unraveling novel regulators of adipocyte differentiation by refining eGRN with STARR-Seq

**Fig. 6:**
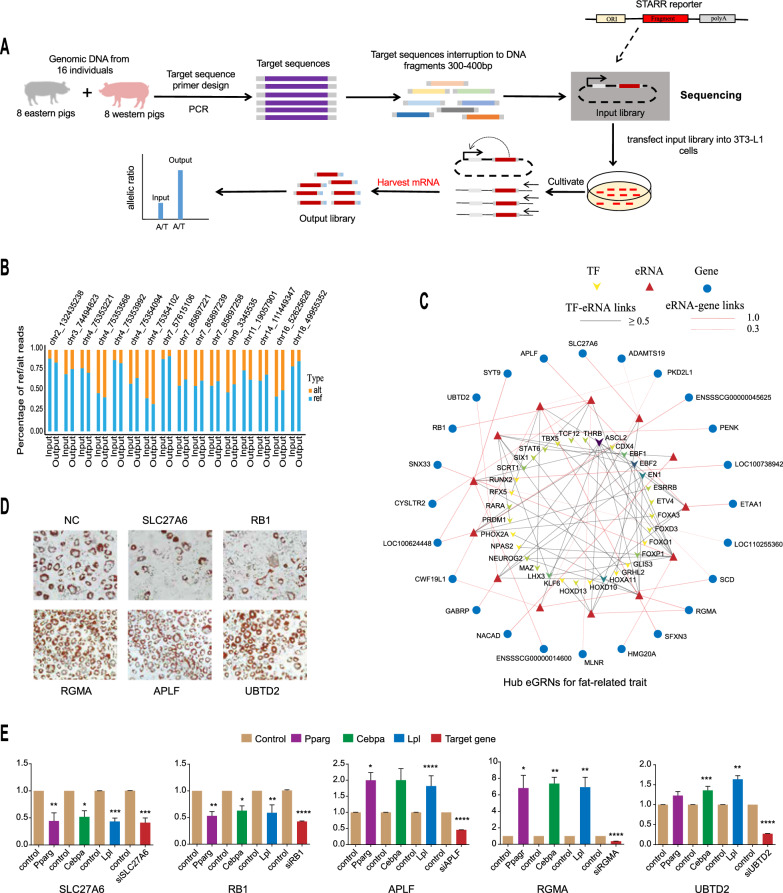
Refining eRNA-mediated gene regulatory networks (eGRN) by self-transcribing active regulatory region sequencing (STARR-Seq) and subsequent identification of functional genes in adipocyte differentiation. **a** The workflow for STARR-seq strategy to detect SNPs influencing DNA activity. **b** Regulatory SNPs identified by STARR-seq that affect enhancer activity. **c** Refined eGRN underlying fat-related traits identified through regulatory SNP screening. **d** siRNA knockdown effects of five potential target genes on adipocyte differentiation assessed by Oil Red O Staining in 3T3-L1 pre-adipocytes. NC represents the blank control. **e** siRNA knockdown effects of potential target genes on marker gene expression during adipocyte differentiation in 3T3-L1 pre-adipocytes. (*P ≤ 0.05; **P ≤ 0.01; ***P ≤ 0.001; ****P ≤ 0.0001)

It is important to note that GWAS associations between genetic loci and traits are often statistical in nature, rather than indicative of causal relationships. Thus, fine-mapping trait-associated genetic loci is pivotal for unraveling the mechanisms of trait inheritance. In our study, we constructed an eGRN through eRNA-transcribing regions that harbor DNA variants with potential pig GWAS signals. To enhance the precision of the eGRN, we subsequently performed capture STARR-Seq experiments, enabling high-throughput screening of enhancer activity (Fig. 6a). Through these experiments, we refined the eGRN by selectively retaining eRNA regions that demonstrated allele-specific enhancer activity. Taking the fat trait-associated eGRN as an example, we exploited a series of criteria to select SNPs within eRNA-transcribing regions for STARR-seq validation. These criteria encompassed SNPs that were detected within the pooled population of eastern and western pigs, characterized by a minimum difference in minor allele frequency (MAF) of 0.3 between the two populations, as well as a MAF exceeding 0.05 in the mixed pool. After applying these criteria (see Additional file 4: Figure S1b), we selected 107 SNPs for STARR-seq assays (see Additional file 19: Table S10), and identified 16 regulatory SNPs that influenced enhancer activity in 3T3 cells (Fig. 6b and see Additional file 20: Table S11). We designated the eRNAs with these regulatory SNPs as hub eRNAs and used them to identify refined eGRN that might play crucial roles in regulating fat-related economic traits (see Additional file 21: Table S12). Within the refined eGRN, we observed 24 potential target genes that could be regulated by eRNAs (Fig. 6c).

To confirm the regulatory role of the refined eGRN on fat-related traits, we performed RNA interference experiments on five randomly selected target genes (*SLC27A6*, *RB1*, *APLF*, *RGMA*, *UBTD2*) in the widely used pre-adipocyte 3T3-L1 cell line derived from mouse embryos, which serves as a popular model for investigating adipocyte differentiation. The mouse *Pparg*, *Cebpa*, and *Lpl* genes, which are widely recognized as master regulators of adipogenesis [86–89], were used as marker genes to assess the impact of the target genes on adipogenesis. Among the five potential target genes, we found that *APLF*, *RGMA*, and *UBTD2* played a negative role in adipogenesis while *SLC27A6* and *RB1* played a positive role (Fig. 6e). Oil staining experiments revealed consistent results (Fig. 6d). It is noteworthy that the *APLF*, *RGMA*, and *UBTD2* genes exhibited higher expression levels in western Duroc pigs that are characterized by high lean meat mass. By comparison, the *SCL27A6* and *RB1* genes showed higher expression levels in eastern ES pigs that are known for their superior fat deposition capacity (Fig. 5d). Therefore, the results of the in vitro experimental validation are highly consistent with the correlation between gene expression and meat-relevant phenotypes, indicating that these genes hold promising potential as important candidate genes for future improvement of pork quality. To summarize, we refined fat eGRN by STARR-seq assays, and further validated the functionality of five genes in adipocyte differentiation using RNA interference experiments. Our findings not only provide potential molecular markers for accelerating pig breeding programs, but also offer a novel eRNA-centric approach for fine-mapping GWAS signals and constructing crucial gene regulatory networks that underlie complex traits.

## Discussion

eRNAs are emerging as a type of critical players in transcriptional regulation, which means that they are particularly relevant for deciphering the genetic basis of complex traits and diseases. In spite of substantial efforts to map eRNAs in various biological contexts, there has been limited research on building eRNA-centric transcriptional regulatory networks and using these networks to pinpoint important genes that potentially affect phenotypes. In this study, by combining whole-transcriptome RNA-Seq-based eRNA profiling, GWAS signals and high-throughput STARR-Seq experiments, we present a novel approach to construct trait-affecting hub eGRN and to identify potential trait-affecting genes. We demonstrate the utility of this approach in dissecting the genetic basis of pork production traits and propose potential fat deposition-affecting genes with evidence on their role in adipocyte differentiation in a cell line model. In light of the exponential growth in whole-transcriptome RNA-Seq data across diverse biological contexts, the ever-expanding repertoire of GWAS loci identified by large-scale population genetic studies (such as UK Biobank), and the rising utilization of STARR-Seq (or other MPRA-based techniques) for studying the cis-regulatory impacts of non-coding DNA variants, we believe that our approach possesses great potential for extensively unraveling the genetic basis underlying a wide spectrum of complex traits and diseases across diverse species.

Biological systems involve a diversity of regulatory processes. To understand the molecular bases that underlie these processes, GRN have been extensively built by different experimental and computational methods. Here, we present a novel approach to construct GRN based on tissue-specific eRNAs that have substantial implications in the formation of muscle and fat-related traits. Compared to conventional gene regulatory networks, our eGRN have directionality, encompassing the intricate regulatory cascade that unfolds from upstream TF to eRNAs, and subsequently extending to downstream target genes. Using muscle and fat as examples, we showed that in our eGRN, many upstream TF of eRNAs are known major regulators that affect tissue development, and the functional annotation of downstream target genes exhibited close relevance to tissue-related biological processes. Notably, the precision and robustness of the eGRN were further increased after we combined the GWAS data and STARR-seq data. As an example, through the identification of eRNAs that harbor cis-regulatory SNPs based on pig GWAS signals, we uncovered hub eRNAs and their potential target genes that influence adipocyte differentiation in 3T3-L1 cells. Although our approach for network construction has proven effective and indicates general applicability, it is important to acknowledge its inherent limitations. For instance, when identifying target genes of eRNAs, we typically refer to previous research [27, 31], which suggests that potential target genes of eRNAs are generally located within a ± 1 Mb region of the eRNA. Nevertheless, distal enhancers persist in regulating gene expression even when located more than ± 1 Mb away from transcription start sites (TSS). Refining the identification of enhancer-gene pairs stands to gain significant advantages from technologies capable of assaying chromatin interactions, such as Hi-C and HiChIP. in addition, employing motif sequence scanning to infer TF binding and the incorporation of TAD constraints to predict downstream target genes, while grounded in fundamental biological principles, may also inadvertently introduce noise into the regulatory relationships within the network. Given the ongoing efforts in producing various types of epigenetic data in a wide range of biological contexts, we anticipate that the integration of transcription factor ChIP-seq data and high-order chromatin structure Hi-C data will further optimize our network construction approach, leading to improved accuracy in identifying both upstream TF and downstream target genes of eRNAs.

Recent years have seen a surge of eRNA-related studies, which have identified eRNAs using high-depth RNA-seq data [28–31, 90]. The low and unstable expression of eRNAs makes high-depth RNA-seq data critical for accurate eRNA identification. In our study, the library size of the RNA-seq data ranged from 38 to 50 million reads per sample, providing a higher depth of RNA-seq data for eRNA identification compared to the study of Carullo et al. [30]. Furthermore, the RNA-seq samples displayed strong reproducibility (Fig. 1c), enabling us to accurately profile an eRNA expression atlas in pigs. The previous understanding of eRNAs was that they were transcribed in a bi-directional manner [91], However, subsequent studies have revealed that some eRNAs are transcribed in a unidirectional fashion [65, 66], which is supported by the results of our study, where we identified different transcriptional patterns of eRNA in tissues and discovered that they have different molecular characteristics and are involved in different biological processes. Although deep learning classification models are used for sequence feature learning, the classification of unidirectional and bidirectional transcribed enhancers remains a challenging endeavor. This ongoing difficulty suggests that the transcription direction of eRNAs may not be only determined by the linear sequence itself, but rather that it may be influenced by complex higher-order chromatin structural dynamics that extend beyond the scope of eRNA sequence analysis.

Transposable elements play a crucial role in shaping the evolution of cis-regulatory elements. An intriguing finding in our study is the significant and specific enrichment of LTR families in TEn. However, it is worth noting that the LTR/ERVK family did not exhibit significant enrichment, which is likely due to insufficient statistical power as a result of its small number of copies in the pig genome. Previous research has established the role of LTR transposons in driving the transcription of lncRNAs in the human and mouse genomes [92]. As a result, we propose that LTR transposons might also play a key role in the transcription of eRNA.

Over the years, there have been many studies on the genetic basis of pig production traits. For example, candidate SNP or genes that affect pig production traits have been identified through GWAS [93–95], by differential gene expression analysis or co-expression network analysis using RNA-seq data from different pig breeds [96–98], and by integrating epigenetic modifications [99, 100]. While these studies have undoubtedly offered valuable insights into the genetic mechanisms that underlie pork production traits, it is crucial to recognize that biological processes operate through intricate, multi-layered networks. Relying solely on the analysis of individual or limited omics data is inadequate to comprehensively unravel the complex genetic regulatory mechanisms that govern these traits. Aligned with this perspective, our study exemplifies an integrative approach that demonstrates the power of exploiting multi-omics data to dissect the genetic basis of pig economic traits.

The functional annotation of genomes has provided a solid foundation for the interpretation of regulatory mechanisms of genetic variations, particularly those in the non-coding regions [101, 102]. Previous studies have shown that tissue-specific cis-regulatory elements enriched for GWAS signals are associated with complex phenotypes [72], highlighting the importance of this type of functional annotation in understanding the genetic basis of phenotypic variations. Here, by comparing eRNA annotation with pig GWAS and QTL data, our results suggest the pivotal role of tissue-specific eRNAs in shaping economic traits in pigs. Beyond pig complex traits, our study also demonstrated that pig muscle- and fat-specific eRNAs can effectively serve as surrogates to decipher the hereditary nature of human muscle- and fat-related phenotypes. This finding further strengthens the utilization of pigs as models for medical research in humans. This is reminiscent of using orthologous-based mouse-derived human open chromatin profiles for understanding the genetic basis of human diseases or phenotypes [37]. Notably, compared to other model species, pigs are generally regarded as anatomically and developmentally more similar to humans, enhancing their relevance as model organisms.

While our systematic study on pig tissue eRNAs provides a valuable approach for understanding complex traits in pigs, it still has certain limitations. For instance, in the identification of eRNAs, we followed the methodology outlined by Carullo et al. [30], excluding known coding gene and non-coding RNA regions based on the reference genome annotation to prevent interference with enhancer transcription signals. In spite of our efforts to leverage annotated genomic files from different databases (RefSeq, UCSC, and Ensembl) for the pig reference genome, the possibility of incomplete annotation remains. Unannotated genes or transcription elements in the pig reference genome are an inevitable objective reality. In addition, when our aim was to screen for eRNAs associated with GWAS hits linked to specific traits, we referred to methodologies applied in previous studies [103, 104], using a 20-kb window to consider the effect of LD of SNPs instead of directly computing LD values between SNPs. However, we recognize that under conditions where direct LD calculations are feasible for the pig population, selecting trait-associated SNPs within eRNAs could yield more precise results. Furthermore, although our study provides the first pig tissue-level eRNA expression atlas and offers a framework for constructing eGRN to dissect meat-related traits in pigs, there is room for optimization. Our focus was on 2-week-old pigs from eastern and western breeds, which overlooked the temporal specificity of eRNA expression at various developmental stages. Future integration of complementary transcriptomic and H3K27ac data across additional developmental time points will enhance the utility of eGRN in unraveling the genetic mechanisms for meat-related traits in pigs.

## Conclusions

In summary, our work not only identified critical eRNAs and their target genes that might contribute to the genetic basis of pork production traits, but also proposed a novel strategy to construct eRNA-centered GRN that pinpoint potential trait-affecting genes. This strategy is broadly applicable for uncovering how genetic components in cis-regulatory elements, such as enhancers, might shape phenotypic diversity in various organisms.

### Supplementary Information

**Additional file 1. **Simulating tissue-specific eRNA elements in the genome 1000 times.

**Additional file 2: Table S1.** The GWAS-associated loci for muscle and adipose tissue-related traits in pigs.

**Additional file 3: Table S2.** The list of primers for enhancer elements used in the self-transcribing active regulatory region sequencing (STARR-seq) system.

**Additional file 4: Figure S1.** Detecting SNP regulatory activity in the self-transcribing active regulatory region sequencing (STARR-seq) system. (**a**) The library correlation in STARR-seq through a correlation analysis of count data in 22 eRNA regions. (**b**) SNP screening protocol for identifying regulatory activity in the STARR-seq system.

**Additional file 5: Table S3.** The siRNA target sequence and RT-qPCR primers.

**Additional file 6: Table S4.** Identified locations of enhancers in each tissue.

**Additional file 7: Figure S2.** Identification methods and functional enrichment analysis of eRNAs. (**a**) Schematic diagram of unidirectional and bidirectional eRNA identification. (**b**) Gene ontology (GO) analysis reveals differential biological process pathways associated with eRNAs with distinct transcriptional directions. GO enrichment analysis was performed based on neighboring genes of eRNAs.

**Additional file 8: Table S5.** Location information of detectable eRNAs in each tissue.

**Additional file 9: Figure S3.** A heatmap revealing the dynamic expression atlas of detectable eRNAs in muscle and adipose tissues across breeds.

**Additional file 10: Figure S4.** Pearson correlation between eRNA expression level and enhancer activity in specific tissue.

**Additional file 11: Figure S5.** Ranked distribution plot of H3K27ac signal density identifies a small subset of super-enhancers. (**a**) Ranked distribution plot of H3K27ac signal density in Duroc fat, Duroc muscle (**b**), ES fat (**c**) and ES muscle (**d**) tissues, along with the number of identified super-enhancers.

**Additional file 12: Table S6.** The expression activity of detectable eRNAs in different tissues.

**Additional file 13: Table S7.** Muscle and fat tissue-specific expression of eRNAs and genes in eastern and western pigs.

**Additional file 14: Figure S6.** Gene ontology (GO) analysis reveals biological process pathways relevant to non-tissue-specific eRNA expression. GO enrichment analysis was performed based on the neighboring genes of eRNAs.

**Additional file 15: Figure S7.** Gene ontology (GO) analysis reveals biological process pathways relevant to genes expressed in a tissue- and breed-specific manner.

**Additional file 16: Table S8.** The genome-wide association study (GWAS) enrichment results of tissue-specific eRNAs in pigs for homologous sequences in humans.

**Additional file 17: Figure S8.** Characteristics and functional enrichment analysis of eRNA target genes in eRNA-mediated gene regulatory networks (eGRN). (**a**) Total number of eRNAs regulating various numbers of target genes. (**b**) Gene ontology (GO) analysis reveals biological functions of target genes regulated by tissue-specific expressed eRNAs in Duroc muscle and Enshi Black (ES) pig fat tissues. (**c**) Expression differences of target genes in muscle-related eGRN between eastern and western pigs (ES vs Duroc).

**Additional file 18: Table S9.** eRNA target genes in eRNA-mediated gene regulatory networks (eGRN) related to muscle and adipose tissue.

**Additional file 19: Table S10.** The list of selected SNPs for STARR-seq experiments.

**Additional file 20: Table S11.** STARR-seq results for SNPs with significant regulatory activity.

**Additional file 21: Table S12.** Regulatory relationships in fat-related hub eRNA target genes in eRNA-mediated gene regulatory networks (eGRN).

## Data Availability

The raw sequence data of STARR-seq used in this paper have been deposited (PRJCA017321) in the Genome Sequence Archive in the BIG Data Center (http://bigd.big.ac.cn/) with the accession code CRA011292.
