# Supplementary material for: Constructing eRNA-mediated gene regulatory networks to explore the genetic basis of muscle and fat-relevant traits in pigs
Source: Genet Sel Evol. 2024 Apr 9;56:28. doi: 10.1186/s12711-024-00897-4 (PMC11003151; doi:10.1186/s12711-024-00897-4)
Supplement: Supplementary file 1 — Additional file 1. Simulating tissue-specific eRNA elements in the genome 1000 times. [file 12711_2024_897_MOESM1_ESM.docx]

Simulate tissue-specific eRNA elements in the genome 1000 times

GWAS=/data/wangchao/method/PIG_animal_DBQTL/Four_association/last_Four_association/20kb/

special_eRNA=/data/wangchao/pig_eRNA/zhao_lab_FAT/other_tissue_RNA/RPM/rep1_2_TSI_eRNA_expression/

for z in $(cat name);

do

for t in Duroc_Fat_specail.bed Duroc_Muscle_specail.bed ES_Fat_specail.bed ES_Muscle_specail.bed;

do

for i in {1..1000}

do

bedtools shuffle -i ${special_eRNA}$t -g susScr11.chrom.sizes -noOverlapping > simulated$i

bedtools intersect -a simulated$i -b ${GWAS}$z > enrich$i

wc -l enrich$i >> $z$t.control.enrich

rm -rf simulated$i enrich$i

done

done

done
