# Supplementary figures and images for: Constructing eRNA-mediated gene regulatory networks to explore the genetic basis of muscle and fat-relevant traits in pigs
Source: Genet Sel Evol. 2024 Apr 9;56:28. doi: 10.1186/s12711-024-00897-4 (PMC11003151; doi:10.1186/s12711-024-00897-4)

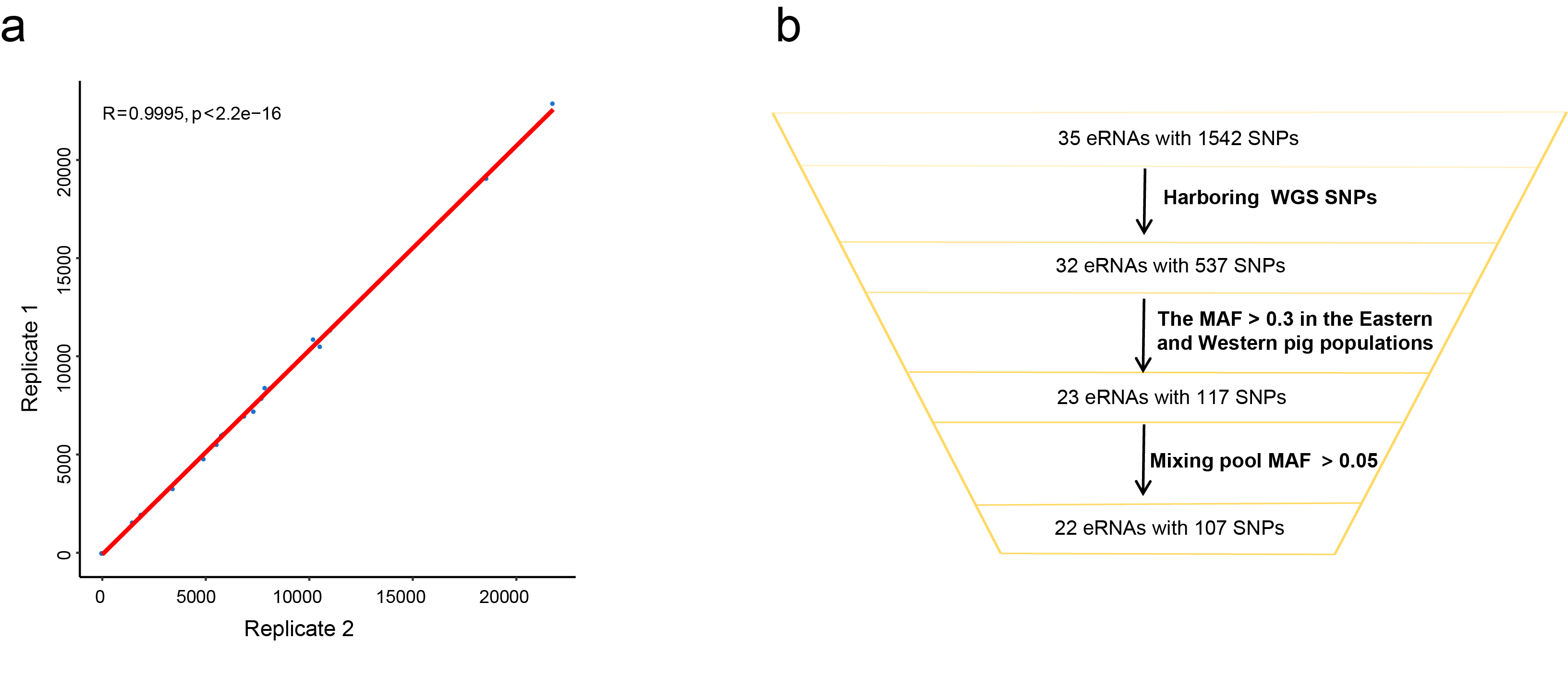

Supplement: Supplementary file 4 — Additional file 4: Figure S1. Detecting SNP regulatory activity in the self-transcribing active regulatory region sequencing (STARR-seq) system. (a) The library correlation in STARR-seq through a correlation analysis of count data in 22 eRNA regions. (b) SNP screening protocol for identifying regulatory activity in the STARR-seq system. [file 12711_2024_897_MOESM4_ESM.tif]

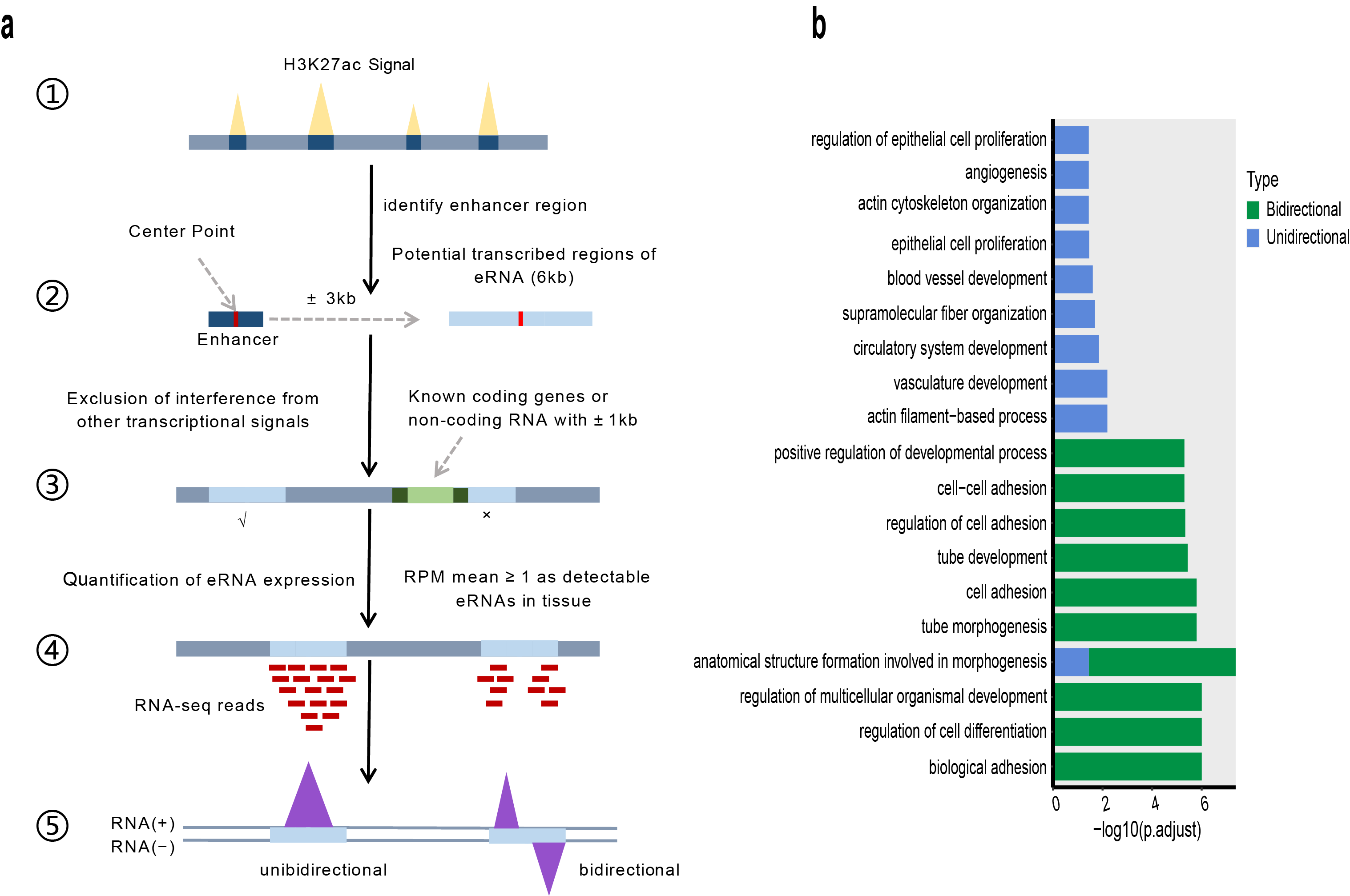

Supplement: Supplementary file 7 — Additional file 7: Figure S2. Identification methods and functional enrichment analysis of eRNAs. (a) Schematic diagram of unidirectional and bidirectional eRNA identification. (b) Gene ontology (GO) analysis reveals differential biological process pathways associated with eRNAs with distinct transcriptional directions. GO enrichment analysis was performed based on neighboring genes of eRNAs. [file 12711_2024_897_MOESM7_ESM.tif]

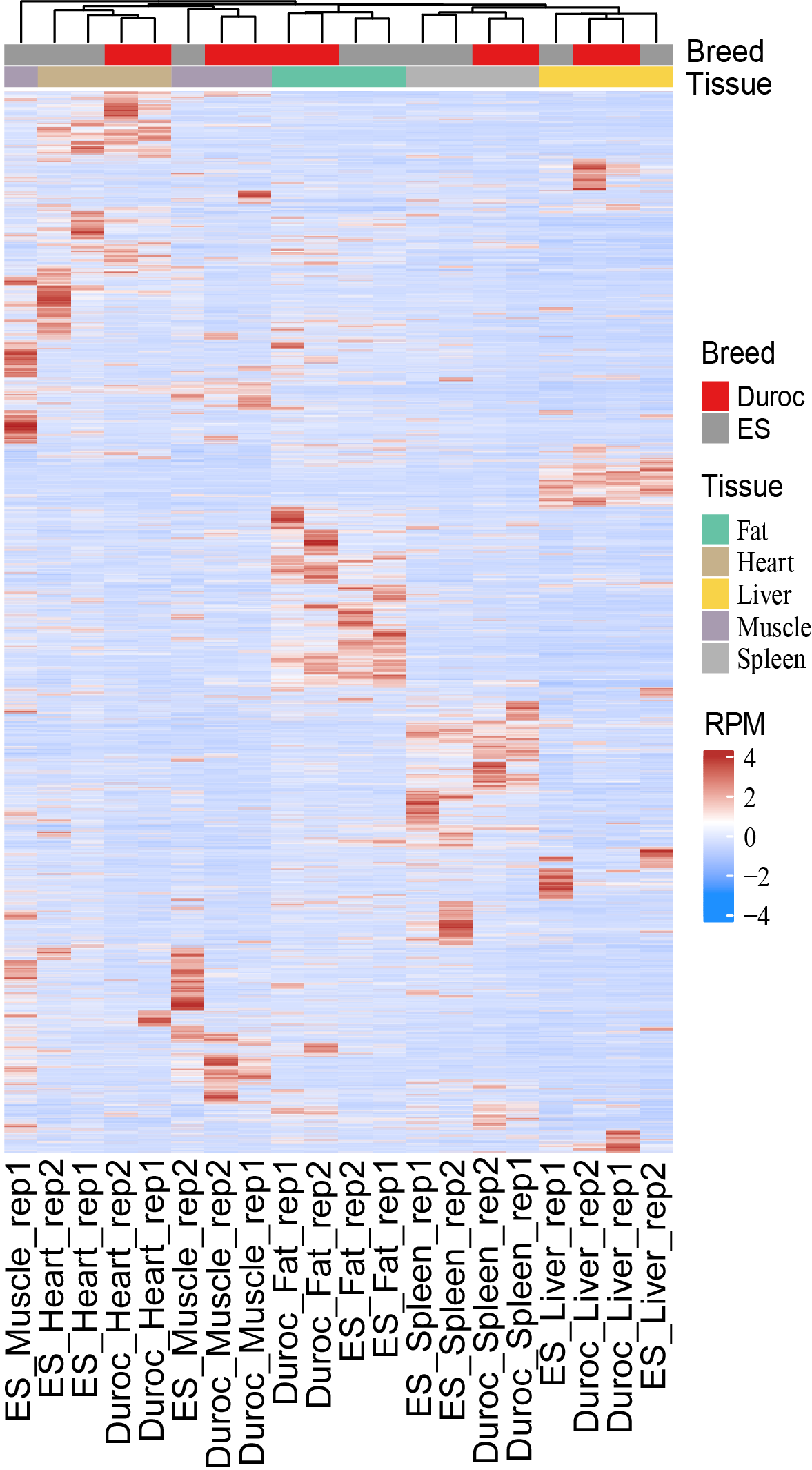

Supplement: Supplementary file 9 — Additional file 9: Figure S3. A heatmap revealing the dynamic expression atlas of detectable eRNAs in muscle and adipose tissues across breeds. [file 12711_2024_897_MOESM9_ESM.tif]

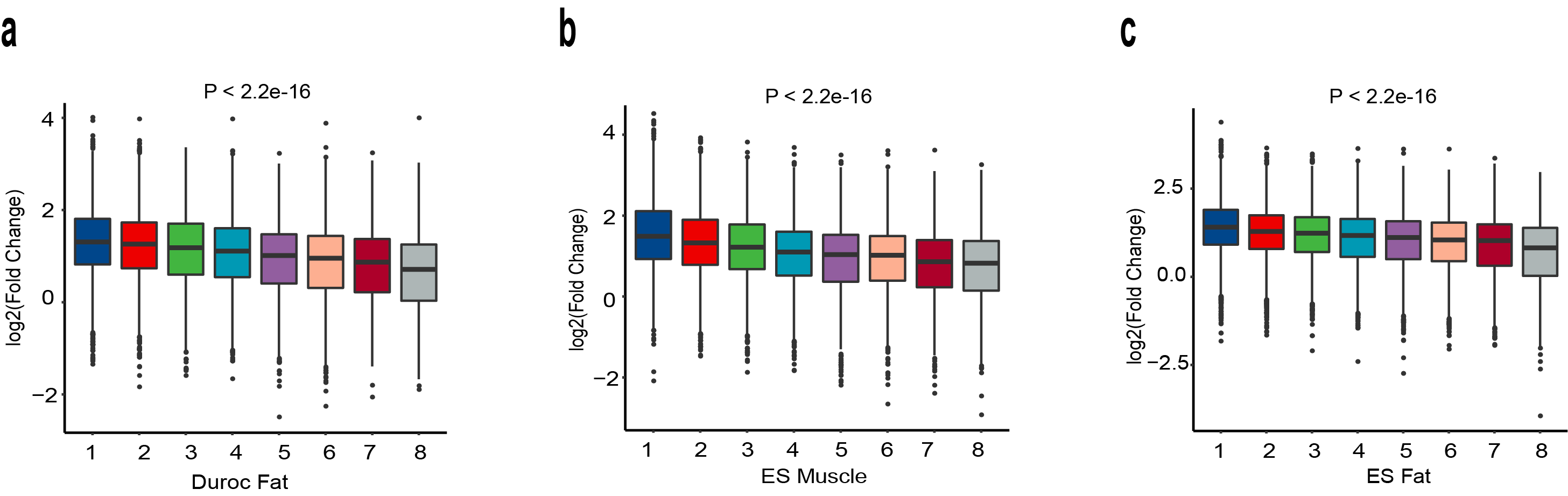

Supplement: Supplementary file 10 — Additional file 10: Figure S4. Pearson correlation between eRNA expression level and enhancer activity in specific tissue. [file 12711_2024_897_MOESM10_ESM.tif]

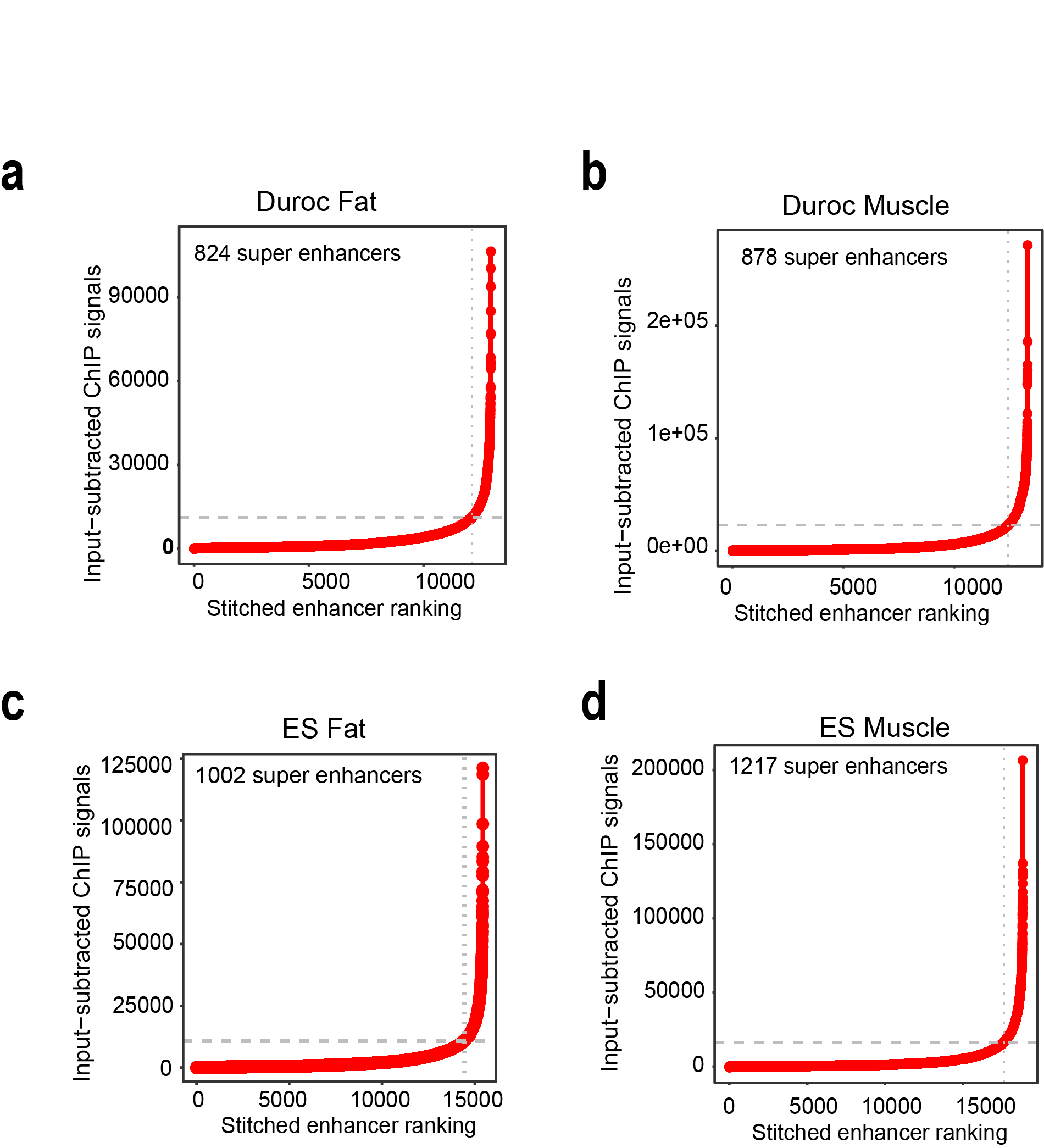

Supplement: Supplementary file 11 — Additional file 11: Figure S5. Ranked distribution plot of H3K27ac signal density identifies a small subset of super-enhancers. (a) Ranked distribution plot of H3K27ac signal density in Duroc fat, Duroc muscle (b), ES fat (c) and ES muscle (d) tissues, along with the number of identified super-enhancers. [file 12711_2024_897_MOESM11_ESM.tif]

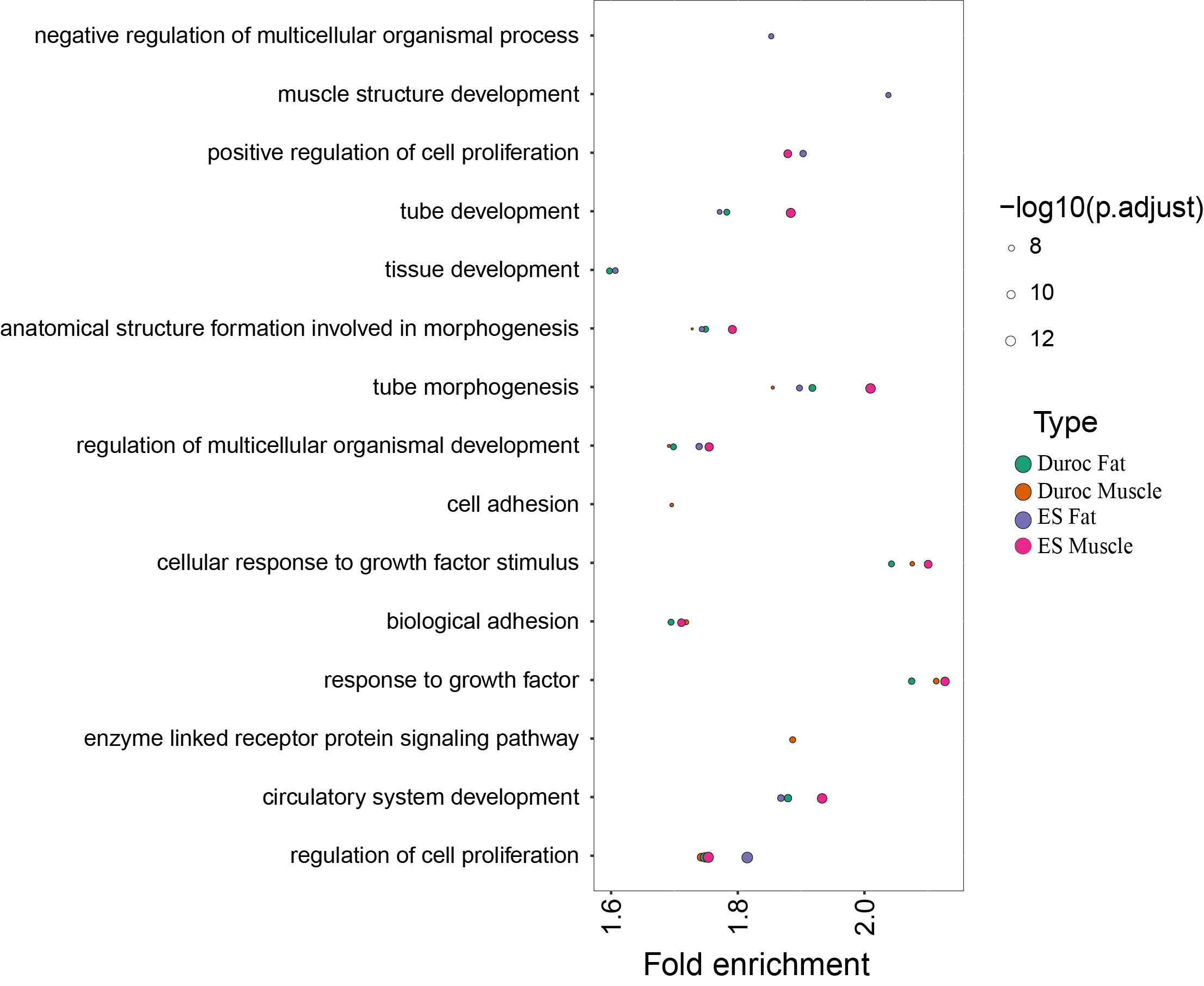

Supplement: Supplementary file 14 — Additional file 14: Figure S6. Gene ontology (GO) analysis reveals biological process pathways relevant to non-tissue-specific eRNA expression. GO enrichment analysis was performed based on the neighboring genes of eRNAs. [file 12711_2024_897_MOESM14_ESM.tif]

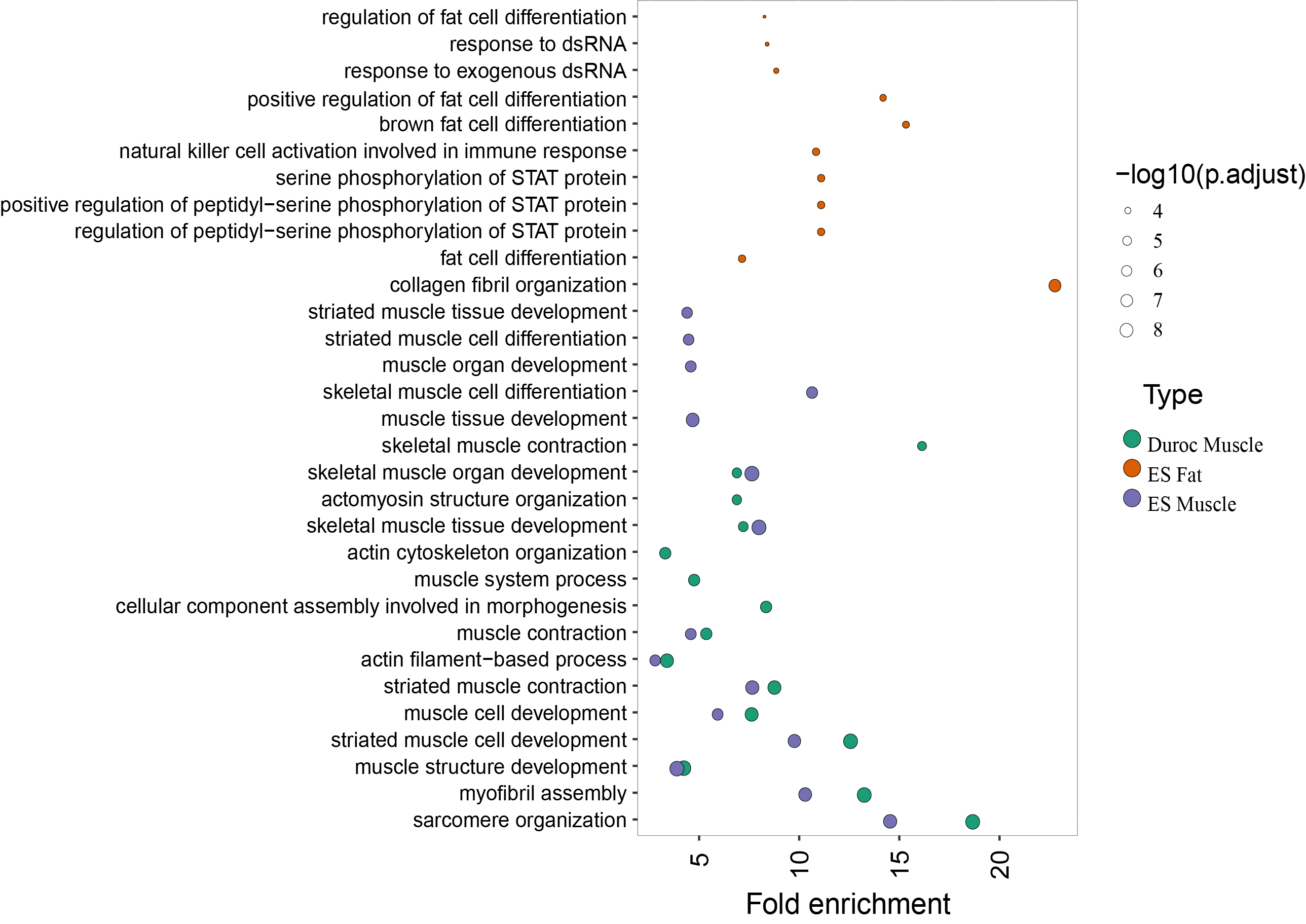

Supplement: Supplementary file 15 — Additional file 15: Figure S7. Gene ontology (GO) analysis reveals biological process pathways relevant to genes expressed in a tissue- and breed-specific manner. [file 12711_2024_897_MOESM15_ESM.tif]

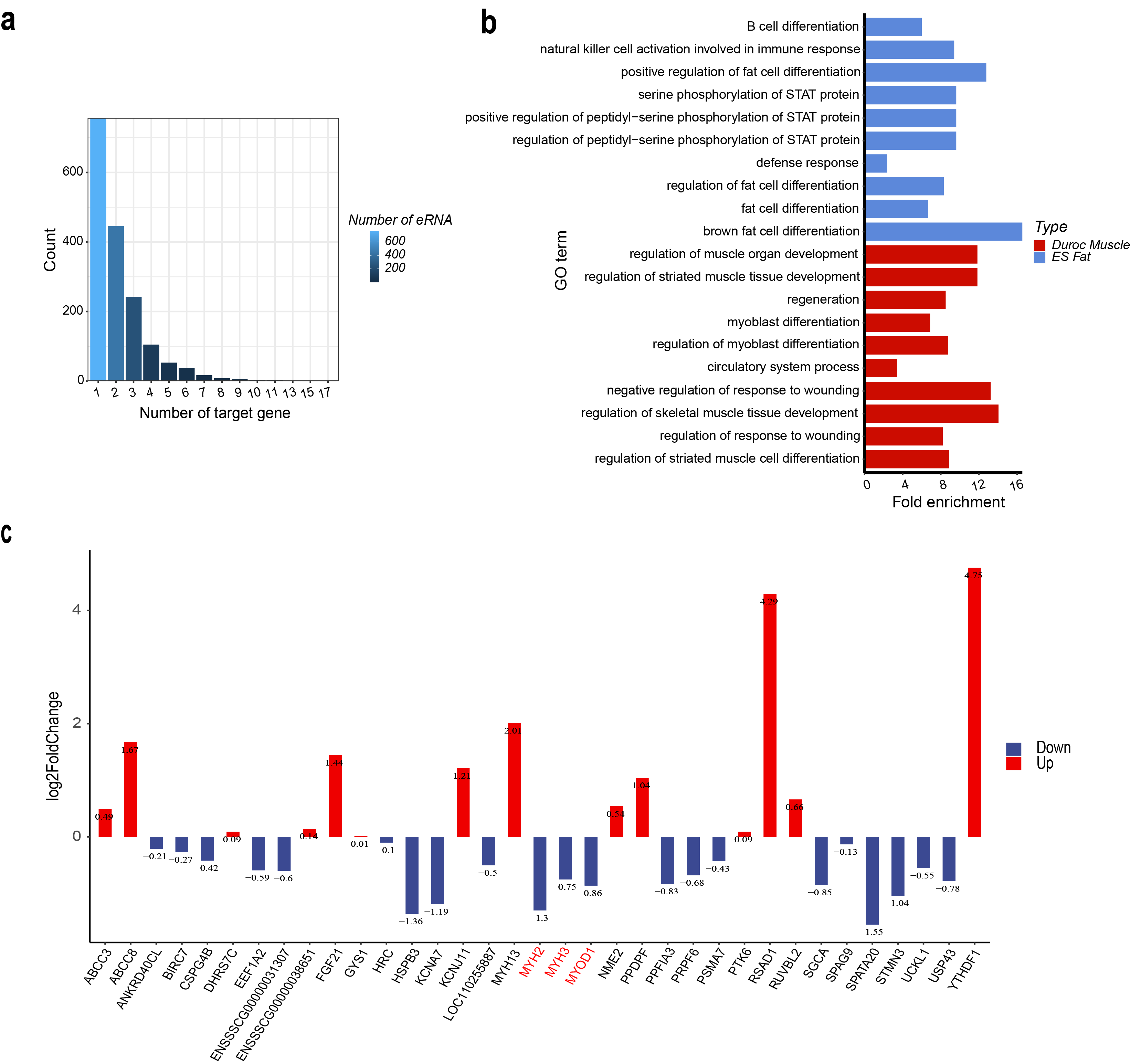

Supplement: Supplementary file 17 — Additional file 17: Figure S8. Characteristics and functional enrichment analysis of eRNA target genes in eRNA-mediated gene regulatory networks (eGRN). (a) Total number of eRNAs regulating various numbers of target genes. (b) Gene ontology (GO) analysis reveals biological functions of target genes regulated by tissue-specific expressed eRNAs in Duroc muscle and Enshi Black (ES) pig fat tissues. (c) Expression differences of target genes in muscle-related eGRN between eastern and western pigs (ES vs Duroc). [file 12711_2024_897_MOESM17_ESM.tif]
